# Association of serum levels of inflammatory cytokines with retinopathy of prematurity in preterm infants

**DOI:** 10.3389/fped.2023.1195904

**Published:** 2024-01-08

**Authors:** Xiao Chun Ling, Pin-Hsuan Huang, Hung-Chi Chen, Yi-Jen Hsueh, Chia-Wen Lee, Reyin Lien, Chien-Chung Lee, Shih-Ming Chu, Kuan-Jen Chen, Yih-Shiou Hwang, Chi-Chun Lai, Ming-Chou Chiang, Wei-Chi Wu

**Affiliations:** ^1^ Department of Ophthalmology, Linkou Chang Gung Memorial Hospital, Taoyuan, Taiwan; ^2^ Center for Big Data Analytics and Statistics, Chang Gung Memorial Hospital, Taoyuan, Taiwan; ^3^ College of Medicine, Chang Gung University, Taoyuan, Taiwan; ^4^ Center for Tissue Engineering, Chang Gung Memorial Hospital, Linkou Branch, Taoyuan, Taiwan; ^5^ Division of Neonatology, Department of Pediatrics, Linkou Chang Gung Memorial Hospital, Taoyuan, Taiwan; ^6^ Department of Ophthalmology, Chang Gung Memorial Hospital, Keelung, Taiwan

**Keywords:** cytokine, retinopathy of prematurity, biomarker, intravitreal injection, anti-VEGF

## Abstract

**Introduction:**

Retinopathy of prematurity (ROP) is a retinal vascular developmental disease associated with risks factors such as supplementary oxygen use or low birth weight/early gestational age. Multiple studies have reported associations between ROP and systemic inflammation. In this study, we investigated serum cytokines associated with ROP development and severity and assessed their applicability as potential biomarkers of ROP.

**Methods:**

This prospective study was conducted at an institutional referral center between 2019 and 2021. To measure the serum levels of 40 inflammatory cytokines in eligible premature patients, we collected their serum samples during the enrollment of patients or the intravitreal injection of anti–vascular endothelial growth factor (VEGF) agents and after 2 and 4 weeks.

**Results:**

Fifty patients were enrolled. In patients with type 1 ROP who received anti-VEGF agents (*n* = 22), the levels of serum intercellular adhesion molecule-1 decreased significantly (*p* < 0.05) at 4 weeks compared with the baseline level, whereas those of serum granulocyte–macrophage colony-stimulating factor increased significantly (*p* < 0.05). In patients with ROP who did not require any treatment (*n* = 14), no significant change was noted in the level of any of the 40 inflammatory cytokines. In control infants without ROP (*n* = 14), the serum levels of tumor necrosis factor-α, interleukin (IL)-15, and IL-12p40 increased significantly (*p* < 0.05) at 4 weeks. The changes in the levels of serum inflammatory cytokines did not vary significantly among the aforementioned three groups. A generalized estimating equation indicated that zone 1 ROP, stage 3 ROP, older postmenstrual age, respiratory distress syndrome, necrotizing enterocolitis, and sepsis were associated with the changes in serum cytokine levels.

**Conclusions:**

Although significant changes (compared with baseline) were observed in the serum levels of certain inflammatory cytokines in patients with type 1 ROP and infants without ROP, no significant difference in cytokine level fluctuations were noted among the three groups. Changes in serum inflammatory cytokine levels may not predict ROP development or severity. Additional comprehensive studies are warranted to establish their definitive role and significance in ROP, emphasizing the need for continued research in this area.

## Introduction

Retinopathy of prematurity (ROP) is a significant retinal vascular developmental disease and a leading cause of infant blindness worldwide (1). Early detection and effective screening of ROP have been shown to reduce the risk of severe vision impairment (2). However, accurately identifying premature infants at risk of ROP remains challenging, as some infants develop ROP despite the absence of known risk factors (3). This has prompted the exploration of novel biomarkers and advanced imaging techniques to enhance ROP risk assessment and guide appropriate treatment.

Systemic inflammation has been implicated in the development of ROP, with studies suggesting associations between ROP and factors such as prenatal inflammation, infections, and elevated cytokine levels (4–9). Cytokines, including interleukins (IL-6, IL-7, IL-8), tumor necrosis factor-alpha (TNF-α), and monocyte chemoattractant protein-1 (MCP-1), play crucial roles in fetal inflammatory responses and angiogenesis (10–12). Elevated cytokine levels in cord blood and vitreous samples of infants with ROP have been observed, indicating their potential as biomarkers for ROP development (13, 14).

While previous studies have mainly focused on cord blood and amniotic fluid samples, the investigation of serum cytokine levels in premature infants may provide valuable insights for early identification of those susceptible to ROP (15–17). Therefore, the purpose of this prospective study is to examine the association between serum cytokines and the development and severity of ROP in premature infants. By elucidating these associations, we can contribute to the understanding of ROP pathogenesis and potentially improve clinical management strategies.

## Materials and methods

### Study population and setting

This prospective study was conducted at the neonatal intensive care unit of Chang Gung Memorial Hospital, an academic tertiary referral center in Linkou, Taiwan, between January 1, 2019, and December 31, 2021. The study enrolled preterm infants who met the following inclusion criteria: birth weight (BW) of ≤1,500 g, gestational age (GA) of ≤32 weeks at birth, supplementary oxygen use, or other systemic risk factors indicating the need for retinopathy of prematurity (ROP) screening, as determined by neonatologists. The exclusion criteria encompassed ophthalmic diseases other than ROP (e.g., congenital glaucoma, Peter's anomaly, persistent fetal vasculature), major congenital malformations or central nervous system defects, and congenital infections (e.g., syphilis, toxoplasmosis, cytomegalovirus, herpes, rubella) or maternal HIV positivity.

The screening protocol for retinopathy of prematurity (ROP) in this study followed the established guidelines of Chang Gung Memorial Hospital. The initial ROP assessment was conducted between 4 and 6 weeks after birth as determined by the neonatologists' referral. Enrollments into the study occurred after obtaining consent from legal guardians and ensuring that both inclusion and exclusion criteria were met. The study was conducted in accordance with the principles outlined in the Declaration of Helsinki and received approval from the Institutional Review Board (IRB) of Chang Gung Memorial Hospital (approval number: 201902088A3). Informed consent was obtained from parents or legal guardians following the study protocol approved by the IRB.

ROP is divided into five stages (Stages 1–5) based on the progression of the disease. Each stage represents a different level of severity, indicating the extent of abnormal blood vessel growth in the retina. Zone is defined as concentric zones (Zone I, II, and III) describing the location of the abnormal blood vessel growth. “Plus disease” is characterized by dilation and tortuosity of the blood vessels within the posterior pole of the eye, indicating the presence of severe and progressive disease process. Anti–vascular endothelial growth factor (VEGF) was intravitreally administered in patients who received a diagnosis of type 1 ROP at follow-up. According to the International Committee for the Classification of ROP, type 1 ROP is defined as (1) any ROP with plus disease in zone I, (2) stage 3 ROP in zone I without plus, and (3) stage 2 or 3 ROP with plus disease in zone II (18). Treatment was not administered for mild ROP—defined as type 2 ROP or a milder type. Type 2 ROP was defined as follows: (1) stage 1 or 2 without plus disease in zone I and (2) stage 3 without plus disease in zone II. Anything milder than type 2 ROP indicated disease severity not reaching that of type 2, such as stage 1 ROP in zone III and stage 2 ROP in zone II. In this study, all type 1 ROP patients were treated with a single intravitreal injection of anti-VEGF agents.

### Data collection

Data on maternal factors, labor and delivery characteristics, and newborn parameters were collected. Details were collected for the following labor, delivery, and newborn characteristics: sex; GA; BW; delivery mode; Apgar scores at 1 and 5 min; postnatal surfactant use; and comorbidities as diagnosed by neonatologists, such as respiratory distress syndrome (RDS), bronchopulmonary dysplasia (BPD), intraventricular hemorrhage (IVH), and necrotizing enterocolitis (NEC). If ROP was diagnosed, we collected data on the corresponding zone, stage, and absence or presence of plus disease in the more severe eye or in any of the eyes if severity was the same between the two eyes.

Sepsis in infants was defined as clinical sepsis syndrome if the infants exhibited the systemic signs of infection or their blood culture was positive for infection. The systemic signs of infection were defined in accordance with the criteria outlined by the systemic inflammatory response system: hypothermia (<36°C) or fever (>38.5°C), tachycardia or bradycardia, and increased respiratory rate and leukocyte count for age (19).

### Cytokine level measurement

**Figure 1:**
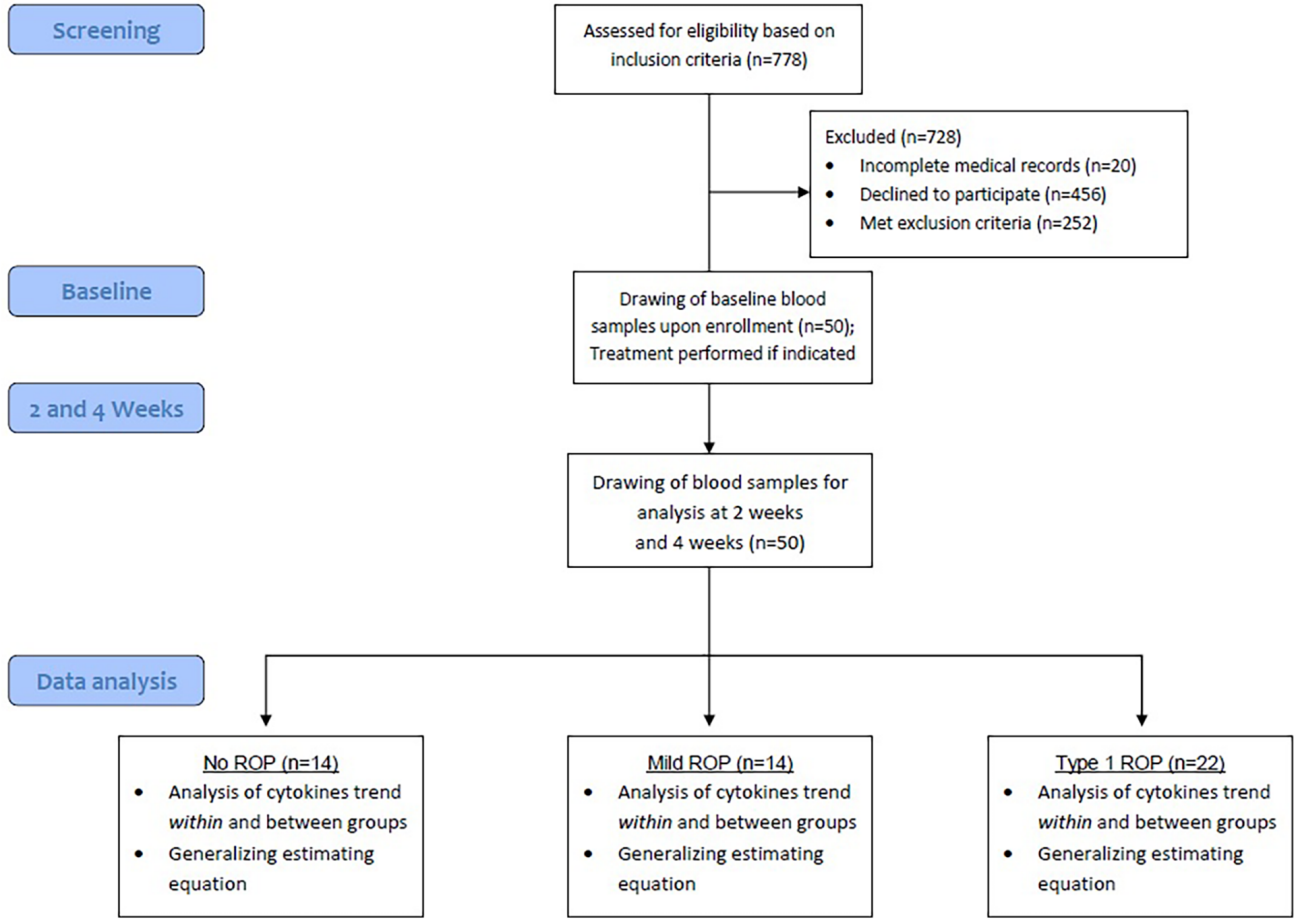
Workflow of the study.

The first blood sample was collected for each patient through venous puncture based on the timeline shown in Figure 1. For type 1 ROP group, bloods were collected just before treatment. Subsequently, blood samples were collected at 2 and 4 weeks during the follow-up period. We used 200 µl of the serum sample for cytokine analysis. Serum samples were immediately centrifuged upon collection, and serum was stored at −80°C until laboratory analysis. All the samples were analyzed in the same research lab, and the cytokine assay was conducted on all samples by the same research staff.

The serum levels of 40 inflammatory cytokines were measured using a commercially available kit (Quantibody Human Inflammation Array 3; RayBiotech, Peachtree Corners, GA, USA). This multiple assay kit was used to simultaneously quantify all 40 cytokines: B-lymphocyte chemoattractant (BLC); eotaxin-1; eotaxin-2; granulocyte colony-stimulating factor (GCSF); granulocyte–macrophage colony-stimulating factor (GMCSF); I-309; intercellular adhesion molecule-1 (ICAM-1); interferon (IFN)-*γ*; IFN-1*α*; IFN-1β; IL-1R*α*; IL-2; IL-4; IL-5; IL-6; IL-6R; IL-7; IL-8; IL-10; IL-11; IL-12p40; IL-12p70; IL-13; IL-15; IL-16; IL-17; monocyte chemoattractant protein (MCP)-1; macrophage colony-stimulating factor (MCSF); monokine induced by IFN-*γ* (MIG); macrophage inflammatory protein (MIP)-1a; MIP-1b; MIP-1d; platelet-derived growth factor-BB; regulated upon activation, normal T cell expressed and secreted (RANTES); tissue inhibitor of metalloproteinase (TIMP)-1; TIMP-2; tumor necrosis factor (TNF)-*α*; TNF-*β*; TNF receptor (TNFR)-1; and TNFR-2. All blood samples were analyzed in duplicate in accordance with the manufacturer's instructions. If the serum cytokine levels fell below the minimum detectable level, they were considered undetectable and therefore, assigned a value of 0 for statistical analysis.

### Statistical analysis

Nonparametric analysis was performed using the *χ*^2^ test for categorical variables and analysis of variance (ANOVA) for continuous variables. Cytokine levels are presented in terms of the mean ± standard deviation values and median with interquartile range values obtained at the three time points (baseline, 2 weeks, and 4 weeks). The level of each serum cytokine at 2 and 4 weeks was compared with its baseline level by using the Wilcoxon signed-rank test. To compare the proportion of changes from baseline in each cytokine level among the three groups, namely no ROP, nontreatment indicated ROP, and type 1 ROP, both parametric ANOVA and nonparametric Kruskal–Wallis tests were used. A generalized estimating equation (GEE) adjusted for the effects of clustered and longitudinal nature of data was used to identify the association between neonatal or ROP factors and serum cytokines levels. Statistical analyses were performed using SAS (version 9.1; SAS Institute, Cary, NC, USA). A *p*-value of <0.05 was considered to be statistically significant.

## Results

### Characteristics of the study population

**Figure 2:**
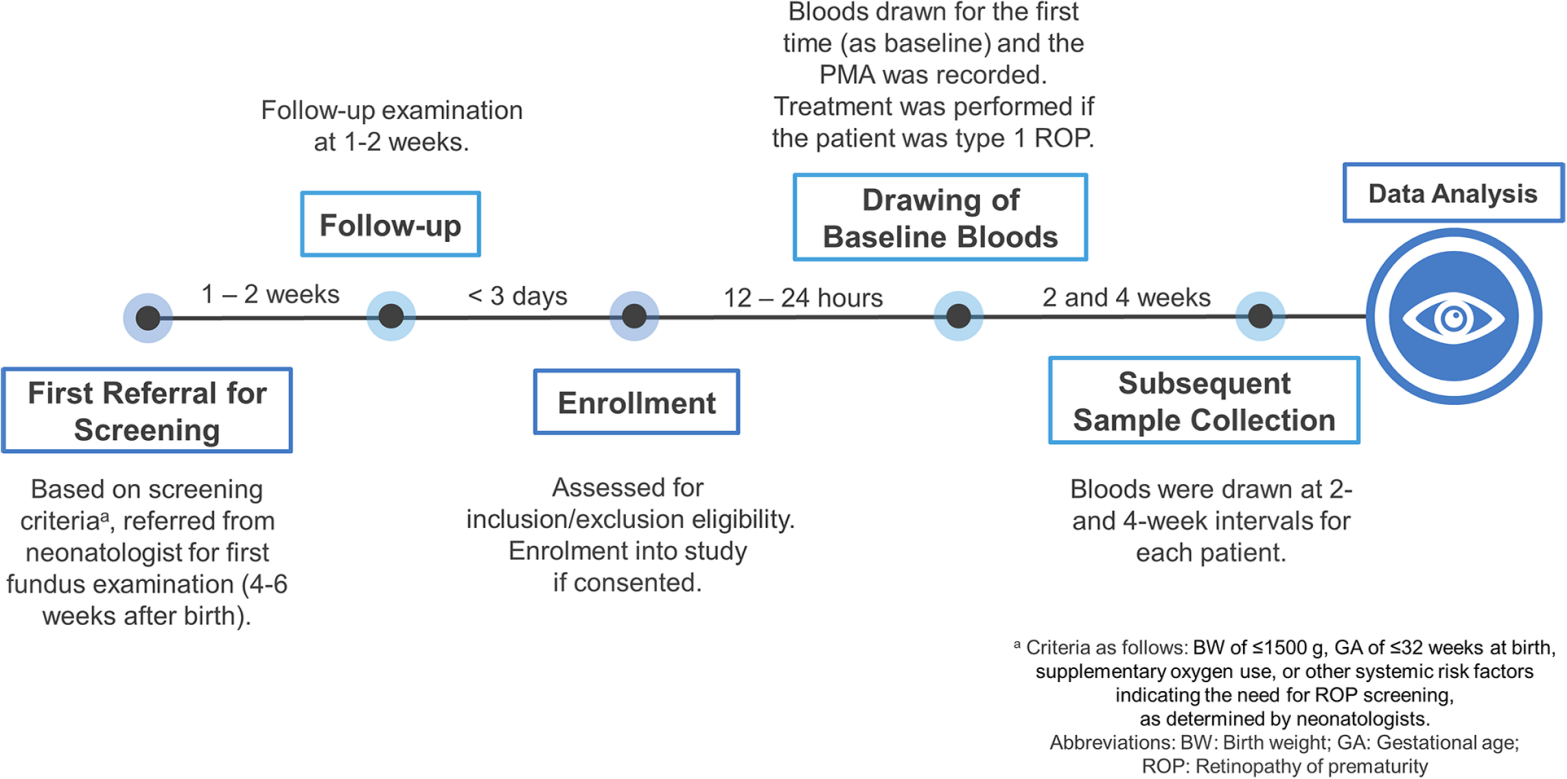
Protocol for study enrollment and sample collection.

The study protocol was summarized in Figure 2. This study included 50 patients, of whom 22 (44%) with type 1 ROP received intravitreal injections of anti-VEGF agents, 14 (28%) did not require any treatment for ROP (mild ROP), and 14 (28%) did not have ROP (control). Table 1 presents the demographic and comorbidity data of the aforementioned three groups. Patients not requiring ROP treatment were diagnosed as having mild ROP (Supplementary Table S1).

**Table 1:** Demographic characteristics of premature infants (*N* = 50).

Variables	No ROP (*n* = 14)	Mild ROP (*n* = 14)	Type 1 ROP (*n* = 22)	*P*-values
Male gender, *n* (%)	7 (50.0)	8 (57.0)	14 (64.0)	*P* = 0.72
Gestational age, weeks (mean ± SD)	31.15 ± 1.72^[A]^	26.68 ± 1.58^[B]^	26.10 ± 2.20^[B]^	*P* < 0.05*
Birthweight, g (mean ± SD)	1,163.14 ± 248.53^[A]^	871.57 ± 206.79^[B]^	846.36 ± 322.03^[B]^	*P* = 0.002*
Baseline post-menstrual age, weeks (mean ± SD)	35.06 ± 1.47	35.69 ± 1.22	36.42 ± 2.19	*P* = 0.0581
Premature Comorbidities				
Sepsis^a^, *n* (%)	3 (21.0)^[A]^	10 (71.0)^[B]^	10 (45.0)^[B]^	*P* = 0.031*
IVH, *n* (%)	9 (64.0)	3 (21.0)	11 (50.0)	*P* = 0.079
RDS, *n* (%)	11 (79.0)^[A]^	14 (100.0)^[B]^	22 (100.0)^[B]^	*P* = 0.037*
NEC, *n* (%)	1 (7.0)	2 (14.0)	2 (9.0)	*P* = 0.86
BPD, *n* (%)	6 (43.0)^[A]^	13 (93.0)^[B]^	20 (91.0)^[B]^	*P* = 0.002*
Surfactant use, *n* (%)	4 (29.0)	6 (43.0)	14 (64.0)	*P* = 0.12

ROP, retinopathy of prematurity; IVH, intraventricular hemorrhage; NEC, necrotizing enterocolitis; RDS, respiratory distress syndrome; BPD, bronchopulmonary dysplasia.

* Statistical significance shown at *p* < 0.05.

[A, B, C]: Inter-group comparisons using *post hoc* test; different (or the same) letters represent significant (or insignificant) statistical differences.

^a^ Defined as the clinical syndrome in which the included infant manifested systemic signs of infection: The presence of at least two of core body temperature (<36 or >38.5), tachycardia or bradycardia, increased respiratory rate and leukocyte count for age.

A total of 7 (50%), 8 (57%), and 14 (64%) patients in the control, mild ROP, and type 1 ROP groups, respectively, were boys. The distribution of sex did not vary significantly among the three groups. GA at delivery was 31.15 ± 1.72, 26.68 ± 1.58, and 26.1 ± 2.20 weeks in the control, mild ROP, and type 1 ROP groups, respectively. BW was 1,163.14 ± 248.53 g in the control group, 871.57 ± 206.79 g in the mild ROP group, and 846.36 ± 322.03 g in the type 1 ROP group. Both GA and BW were significantly larger in the control group than in the ROP groups (*p* < 0.05). No significant difference was noted in PMA, based on which the baseline level was determined, among the three groups (*p* = 0.0581), indicating a well-matched baseline.

In terms of comorbidities, the proportions of infants with NEC, infants with IVH, and cases of surfactant use were comparable among the three groups. The proportions of the patients with RDS, BPD, and sepsis were higher in the mild and type 1 ROP groups than in the control group (*p* < 0.05). Most patients with ROP had zone 2 ROP, with 92.9% and 81.8% of them having mild and type 1 ROP, respectively. Furthermore, 18.2% of the patients with type 1 ROP were diagnosed as having zone 1 ROP. Approximately 95.5% patients in the type 1 ROP group were diagnosed as having stage 3 ROP compared with 14.3% in the mild ROP group. Only the type 1 ROP group included patients with ROP plus disease (90.9%).

### Serum levels of inflammatory cytokines

The serum levels of all the 40 cytokines were measured at baseline, 2 weeks, and 4 weeks for each patient in the three groups (Supplementary Tables S2–S4). The tables list the median and the interquartile range and the mean and standard deviation of all measured serum cytokine for the three groups. Table 2 presents the serum cytokines that showed significant changes from baseline levels at either 2 or 4 weeks.

**Table 2:** Serum cytokines with significant changes from baseline levels at either 2 weeks or 4 weeks in each group.

Serum cytokines/time	Type 1 ROP (*N* = 22)		Mild ROP (*N* = 14)		No ROP (*N* = 14)	
	Median (range), pg/ml	*P*-value	Median (range), pg/ml	*P*-value	Median (range), pg/ml	*P*-value
GCSF						
Baseline	19.05 (4.85–49.40)	N/A	21.15 (8.48–29.53)	N/A	17.73 (5.24– 26.97)	N/A
2 weeks	8.23 (4.00–22.72)	**0**.0035*	16.20 (7.80–25.91)	0.9515	10.91 (5.47–24.92)	0.6698
4 weeks	11.58 (3.07–22.20)	0.1756	10.77 (7.31–20.29)	0.5416	11.60 (4.37–30.68)	0.8552
GMCSF						
Baseline	50.82 (0.00–80.44)	N/A	72.10 (41.09–100.47)	N/A	46.43 (9.75–63.80)	N/A
2 weeks	63.23 (10.22–104.45)	0.2413	51.39 (30.64–111.36)	0.9515	43.11 (31.10–59.27)	1.0000
4 weeks	87.18 (39.94–116.00)	**0**.0425*	89.54 (54.92–119.84)	0.4631	38.37 (14.42–60.27)	0.7148
ICAM						
Baseline	3,340.34 (2,881.95–3,682.14)	N/A	3,183.31 (2,841.07–3,580.39)	N/A	3,115.91 (2,609.25–3,303.06)	N/A
2 weeks	3,316.03 (2,878.72–3,671.71)	0.3843	3,146.19 (2,878.19–3,375.04)	0.8552	2,951.35 (2,727.46–3,530.23)	0.7148
4 weeks	3,308.47 (2,795.24–3,557.26)	**0**.0467*	2,947.40 (2,511.12–3,328.47)	0.0906	3,097.31 (2,595.47–3,463.40)	0.9515
TNFR2						
Baseline	7,383.83 (6,771.80–8,371.63)	N/A	7,200.44 (6,309.92–8,558.88)	N/A	7,302.48 (6,063.24–8,191.01)	N/A
2 weeks	6,862.43 (6,038.01–7,897.22)	**0**.0246*	7,279.81 (7,151.04–8,056.21)	0.8552	7,541.94 (5,883.88–7,908.38)	0.8552
4 weeks	6,524.56 (5,412.89–7,829.56)	**0**.0467*	7,279.81 (7,151.04–8,056.21)	0.1937	7,042.34 (6,512.13–7,792.94)	0.8552
IL12p40						
Baseline	14.97 (7.26–20.25)	N/A	9.05 (4.79–18.19)	N/A	11.48 (5.42–25.99)	N/A
2 weeks	12.79 (4.69–17.68)	0.1951	17.33 (10.54–25.20)	0.2676	17.29 (9.02–25.63)	0.3910
4 weeks	15.27 (7.46–22.98)	0.1342	17.31 (7.93–27.26)	0.3258	22.44 (13.63–27.73)	**0**.0419*
IL15						
Baseline	1.43 (0.69–4.00)	N/A	1.28 (0.62–2.16)	N/A	0.99 (0.43–1.58)	N/A
2 weeks	1.58 (0.73–4.23)	0.9599	0.62 (0.25–0.87)	0.8926	1.01 (0.38–2.27)	0.5016
4 weeks	2.08 (0.66–4.53)	0.9625	1.48 (0.67–3.41)	0.3258	1.22 (0.62–3.17)	**0**.0295*
IL12p40						
Baseline	14.97 (7.26–20.25)	N/A	9.05 (4.79–18.19)	N/A	7,302.48 (6,063.24–8,191.01)	N/A
2 weeks	12.79 (4.69–17.68)	0.1951	17.33 (10.54–25.20)	0.2676	7,541.94 (5,883.88–7,908.38)	0.8552
4 weeks	15.27 (7.46–22.98)	0.1342	17.31 (7.93–27.26)	0.3258	7,042.34 (6,512.13–7,792.94)	0.8552
IL5						
Baseline	12.23 (7.94–21.93)	N/A	12.35 (8.69–17.34)	N/A	7.28 (4.13–14.39)	N/A
2 weeks	13.48 (6.55–24.05)	0.7897	10.63 (3.24–15.48)	0.5830	10.57 (5.76–15.47)	**0**.0353*
4 weeks	16.73 (11.99–45.26)	0.2870	14.05 (8.59–25.78)	0.6257	9.84 (5.58–18.55)	0.2439
TNF*α*						
Baseline	3.96 (1.29–6.57)	N/A	3.89 (2.71–4.83)	N/A	2.96 ± 3.59	N/A
2 weeks	4.25 (1.45–7.00)	0.6261	3.77 (2.11–5.56)	0.6698	3.50 ± 3.64	0.3575
4 weeks	3.73 (2.53–7.24)	0.9375	5.75 (3.60–7.13)	0.2166	6.09 ± 9.91	**0**.0479*

*P*-value was evaluated by Wilcoxon signed-rank test (compared to baseline).

* Statistical significance shown at *p* < 0.05.

In the type 1 ROP group (Table 2), the serum level of ICAM-1 decreased significantly (*p* < 0.05) at 4 weeks compared with the baseline level. A significant and continual reduction was noted in the level of TNFR-2 at 2 weeks (median, 6,862.43 pg/ml; interquartile range, 6,038.01–7,897.22 pg/ml; *p* = 0.0246) and 4 weeks (median, 6,524.56 pg/mL; interquartile range, 5,412.89–7,829.56 pg/ml; *p* = 0.0025) compared with the level at baseline. By contrast, the serum level of GM-SCF increased significantly at 4 weeks (median, 87.18 pg/m; interquartile range, 39.94–116.00 pg/ml; *p* = 0.0425) compared with the baseline level (median, 50.82 pg/ml; interquartile range, 0.00–80.44 pg/ml). The serum level of GCSF in the type 1 ROP group decreased significantly at 2 weeks (median, 8.23 pg/ml; interquartile range, 4.00–22.72 pg/ml; *p* = 0.0035) but not at 4 weeks.

In the mild ROP group (Table 2), no significant change (from baseline) was noted in the serum level of any of the 40 cytokines at 2 or 4 weeks. In the control group (Table 2), the serum level of IL-5 increased significantly at 2 weeks (median, 87.18 pg/ml; interquartile range, 39.94–116.00 pg/ml; *p* = 0.0425). However, the serum levels of IL-15, IL-12p40, and TNF-α increased significantly only at 4 weeks.

### Comparison of the changes in the serum levels of inflammatory cytokines among the three groups

We investigated whether the changes (from baseline) in the serum levels of all 40 inflammatory cytokines varied across the groups (Supplementary Table S5). No significant difference was noted among the three groups in the changes in serum levels at 2 or 4 weeks. Therefore, no distinct change or fluctuation in the serum level of any of the 40 cytokines predicted the development or severity of ROP.

### Factors associated with the changes in the serum levels of inflammatory cytokines

We used a GEE to investigate the association of demographics, ROP, and comorbidities with the changes in the serum levels of inflammatory cytokines (Supplementary Table S6). The following inflammatory cytokines that exhibited significant changes in their serum levels were included in the model for comparison: GCSF, GM-CSF, IL-12p40, IL-15, ICAM-1, IL-5, TNF-α, and TNFR2.

Table 3 lists factors that were significantly associated with the changes in serum cytokine levels. At 2 weeks, zone 1 ROP was associated with increases in the serum levels of GM-CSF (*p* = 0.0184) and IL-5 (*p* = 0.0064). RDS was associated with an increase in the serum level of IL-15 (*p* = 0.0437) at 2 weeks. Furthermore, reductions in the serum levels of inflammatory cytokines at 2 weeks were associated with certain factors. Larger baseline PMA at treatment was associated with significant reductions in the serum levels of ICAM-1 (*p* = 0.0394) and TNFR2 (*p* = 0.0044) at 2 weeks. Both zone 1 and stage 3 ROP were associated with a reduction in the serum level of TNFR2 (*p* = 0.0350 and *p* = 0.0290, respectively). Zone 1 ROP was associated with a reduction in the serum level of ICAM-1 (*p* = 0.0171) at 2 weeks.

**Table 3:** Summary of factors significantly associated with serum cytokines changes according to GEE analysis.

Time/factors	Increased levels of serum cytokines	Decreased levels of serum cytokines
Week 2		
Zone 1 ROP	GMCSF (*p* = 0.0184), IL-5 (*p* = 0.0064)	TNFR2 (*p* = 0.0350)
Stage 3 ROP	–	TNFR2 (*p* = 0.0290)
RDS	IL-15 (*p* = 0.0437)	–
Larger PMA (weeks)	–	TNFR2 (*p* = 0.0044), ICAM (*p* = 0.0394)
Week 4		
Zone 1 ROP	–	ICAM (*p* = 0.0168), TNFR2 (*p* < 0.0001)
RDS	–	ICAM (*p* < 0.0001)
Sepsis	–	ICAM (*p* = 0.0457)
NEC	–	IL-15 (*p* = 0.0383), IL-5 (*p* = 0.0485), TNFα (*p* = 0.0325)

GEE, generalized estimating equation; ROP, retinopathy of prematurity; NEC, necrotizing enterocolitis; RDS, respiratory distress syndrome; BPD, bronchopulmonary dysplasia.

No risk factor was significantly associated with elevated serum levels of inflammatory cytokines at 4 weeks. However, we identified factors that were significantly associated with reduced serum levels of inflammatory cytokines at 4 weeks. Zone 1 ROP was associated with reductions in the serum levels of ICAM-1 (*p* = 0.0168) and TNFR2 (*p* < 0.0001) at 4 weeks. A reduction in the serum level of ICAM-1 was associated with RDS (*p* < 0.0001) and sepsis (*p* = 0.0457). Furthermore, a diagnosis of NEC was significantly associated with reductions in the serum levels of IL-5 (*p* = 0.0485), IL-15 (*p* = 0.0383), and TNF-α (*p* = 0.0325) at 4 weeks.

## Discussion

In this prospective study, we measured the serum levels of 40 inflammatory cytokines in the type 1 ROP, mild ROP, and control groups. The serum levels of various inflammatory cytokines exhibited significant changes (compared with the baseline levels) in the type 1 ROP and control groups. The mild ROP group did not exhibit significant changes (from baseline) in the serum level of any of the 40 cytokines at any time point. The changes in the serum levels of the 40 inflammatory cytokines did not vary significantly across the groups. This finding indicated that the changes in serum cytokine levels from baseline could not predict the development or severity of ROP. GEE results revealed that zone 1 ROP, stage 3 ROP, older baseline PMA, RDS, NEC, and sepsis were associated with increases or decreases in the serum levels of GM-CSF, IL-5, IL-15, ICAM-1, TNF-α, and TNFR-2 in premature infants.

Despite recent advances in medical research (20), clinical biomarkers that can facilitate the early diagnosis or timely management of ROP remain to be identified. Under similar clinical conditions, how and why ROP progresses to a more severe stage in some infants but regresses in other infants remains unknown. Systemic factors were postulated to be responsible for this phenomenon. Thus, various systemic predictors of ROP, such as neonatal illness severity scores, have been identified (21, 22). Many biomarkers associated with ROP and systemic conditions have been extensively researched, but their clinical application depends on the accessibility and availability of rapid detection methods. Measuring serum cytokine levels is feasible and can be used to predict ROP severity and systemic conditions. For instance, our findings suggest that decreased levels of certain cytokines, such as ICAM-1, may be indicative of zone 1 ROP, sepsis, and RDS. However, further studies are necessary to validate the utility of these cytokines as biomarkers.

Compared with baseline findings, significant changes were observed in the levels of certain cytokines at 2 or 4 weeks in the study groups. Cytokines are involved in immune cell recruitment and activation, angiogenesis regulation, cell proliferation, and apoptosis (23, 24). Depending on the target cell and timing of action, cytokines can exert pro- or anti-inflammatory effects. In their population study, Sood et al. (2010) observed a coordinated pattern change in the levels of six inflammatory markers and two growth factors in ROP (25). Moreover, they reported more prominent changes in blood samples collected during the late postnatal period than in those collected during the early postnatal period (25). In this study, we analyzed changes in the serum levels of inflammatory cytokines over a month of ROP treatment or screening to adjust for the bias resulting from the measurement of cytokine levels only at a single time point. Our findings are representative of real-world conditions wherein systemic cytokine levels fluctuate in a manner consistent with the complex interplay between disease activity and protein function.

Certain cytokines, including GCSF, GMCSF, ICAM-1, TNF-α, IL-5, IL-15, and IL-12p40, showed significant changes at 2 or 4 weeks compared to baseline levels. GCSF, a regulator of hematopoiesis and immunity, induces angiogenesis and upregulates VEGF production indirectly by increasing the synthesis of IGF-1 molecules (26–29).. The vitreous level of GCSF was significantly higher in infants with ROP than in those without ROP (30). After the intravitreal injection of anti-VEGF agents, the serum levels of GCSF decreased gradually in our type 1 ROP group. GMCSF, a similar growth factor, facilitates immune cell maturation and cytokine production (31). The role of GMCSF in ROP is not yet studied extensively, but inflammatory mediators are implicated in ROP risk (3, 8). TNFR2 contributes to neural survival and vascular development (32, 33). In our study, the serum level of TNFR2 decreased significantly in the type 1 ROP group at 4 weeks. A possible explanation for this finding is that TNFR2 exerts a protective effect on the retina during the early stage of ROP, which leads to the high expression and secretion of TNFR2. This, in turn, promotes hypoxia-induced revascularization in patients with ROP (33).

Our findings revealed significant increases (compared with baseline levels) in the serum levels of IL-12p40, IL-15, and TNF-α in the control group at 4 weeks. However, GEE revealed no significant association between the 40 inflammatory cytokines and the presence of ROP or comorbidities at week 4. Therefore, the observed increase in the cytokine levels of the control group may be attributed to other factors, such as negative feedback mechanisms against intrinsic inflammatory processes, changes in medication or treatment regimens, and other systemic conditions (34–36). Future studies are warranted to investigate the mechanisms underlying our observations.

The comparison of the changes in serum cytokine levels among the three groups did not reveal a discernible pattern, indicating that the magnitude of changes in systemic cytokine levels is not a reliable parameter for differentiation among the three groups. However, certain cytokines that would exhibit significant changes might not have been included in the current analysis; these cytokines would still contribute to the onset and progression of ROP. Thus, these cytokines should be investigated as potential treatment targets. Although systemic cytokine changes alone may not serve as a reliable predictor of ROP risk, the identification of specific cytokines that are significantly altered may still hold promise for advancing our understanding and management of ROP.

Correlation analysis revealed significant associations between certain ROP or comorbidities and the changes in the serum levels of inflammatory cytokine at 2 and 4 weeks. Zone 1 ROP was associated with most of the changes in the serum levels of cytokines, including increases in GM-CSF and IL-5 levels and decreases in ICAM-1 and TNFR2 levels. This finding implies that hematopoietic factors (e.g., GM-CSF and IL-5; also known as *β* common chain cytokines) involved in regulating the growth and differentiation of immune cells (37) may contribute to inflammatory conditions associated with severe ROP. In an *in vivo* study, these cytokines were demonstrated to regulate various inflammatory responses and contribute to chronic inflammation (37). Although we found no association between IL-5 level and ROP, elevated IL-15 levels were found to be significantly associated with RDS. Agouridakis et al. (2002) reported a positive correlation between the serum level of IL-15 and the severity of acute RDS (assessed using the Acute Physiology and Chronic Health Evaluation II score). This finding suggests that the evaluation of IL-15 level can help screen patients at risk of RDS, thus facilitating the estimation of ROP risk, given a likely correlation between RDS and ROP (38, 39).

Our findings may suggest a protective role of TNFR2 against ROP. At week 2, the reduction in the serum level of TNFR2 was correlated with zone 1 and stage 3 ROP. This finding is consistent with those of studies reporting the role of TNFR2 in promoting hypoxia-induced revascularization without increasing pathological neovascular tufts (33). TNFR2 exhibited combined anti-inflammatory and neuroprotective properties (40, 41). The reduced (from baseline) serum level of ICAM-1 at week 4 was significantly associated with zone 1 ROP, RDS, and sepsis in our patients. This finding may be attributed to the significant upregulation of ICAM-1 at the initial stages of systemic inflammation. The increased level of ICAM-1 facilitated the progression of inflammation before the reduction in ICAM-1 level following the treatment of ROP or the control of systemic diseases (42, 43).

Significant reductions in the serum levels of IL-15, IL-5, and TNF-α at week 4 were correlated with NEC. This finding indicates that the levels of these cytokines are elevated at baseline in patients with NEC. Increased TNF-α levels have been reported in various models of NEC (44). To the best of our knowledge, no study has reported a direct association of IL-5 or IL-15 with NEC; nonetheless, recent studies have indicated an association between the expression of inflammatory genes related to the interleukin pathway and NEC in neonates (45, 46). Although the precise roles of these interleukins are currently unknown, the expression of IL-5- or IL-15-related inflammatory genes and the development of NEC in neonates may be associated. Further studies are necessary to fully comprehend the nature and significance of this association.

The Quantibody Human Inflammation Array and traditional Enzyme-Linked Immunosorbent Assay (ELISA) are both methods for detecting and quantifying inflammatory factors in biological samples. However, they have different advantages and limitations. Quantibody can measure multiple analytes at once, which increases the throughput, reduces the sample volume requirement, and lowers the cost per assay. Traditional ELISA, on the other hand, can only measure one analyte at a time, but it is a more established and widely used technique for measuring specific analytes in a more focused way. Therefore, the choice between them depends on the research or diagnostic objectives (47).

This study has several limitations. The levels of cytokines may vary considerably among individuals. Moreover, medications used in neonatal care, genetics, steroid administration, fetal growth retardation, early or late onset infections, nutritional status and environmental factors may affect cytokine levels. To minimize this variability, we measured cytokine levels in individuals over time. Nevertheless, other unaccounted factors, which were difficult to control at this stage, may require stratification in future studies. Although the difference was not statistically significant, the proportion of the patients with sepsis was higher in the mild ROP group than in the type 1 ROP group. This observation can be attributed to the clinical criteria used to define sepsis in our study, which reflected real-world situations but might have affected the findings. The small sample size of the study may have impacted the statistical power, which can limit the ability to detect statistically significant associations. In addition, the willingness of parents to have their infants participate in the study, which involved biweekly blood draws for premature infants, may have influenced the composition of the ROP groups and might have led to a higher proportion of patients with type 1 ROP. However, the use of a GEE model addresses this concern by considering the within-subject correlation and maximizing the information available from the data. It is important to note also although GEE model helps in adjusting for confounders, it cannot completely eliminate the impact of different characteristics, such as GA and BW, on the results. Interpretation of the findings should still consider the potential influence and based on real clinical situation. In addition, the serum levels of cytokine may not always accurately reflect the degree of inflammation in target organs and thus may not provide a complete overview of immune processes involved in ROP (48). Nevertheless, monitoring gradual changes in the serum levels of cytokines may provide valuable insights into the complex interplay between immune processes and ROP pathogenesis. Nonetheless, these associated serum cytokines can only serve as viable biomarkers if we can enhance our understanding of quantification data and establish a clear demarcation between normal and abnormal levels in future studies.

## Conclusions

Our findings revealed significant changes in the serum levels of certain inflammatory cytokines in patients with type 1 ROP and infants without ROP. However, no significant difference was noted in the changes in cytokine level among the three groups, suggesting that this parameter (changes in the serum levels of inflammatory cytokines) by itself cannot predict the development or severity of ROP. Additional studies are needed to investigate the association between the serum levels of inflammatory cytokines and the development or severity of ROP and to identify the clinical applications of these cytokines.

## Data Availability

The original contributions presented in the study are included in the article/**Supplementary Material**, further inquiries can be directed to the corresponding authors.

## References

[1] Hellstrom A Smith LE Dammann O. Retinopathy of prematurity. Lancet. (2013) 382:1445–57. 10.1016/S0140-6736(13)60178-6 23782686 PMC4389630

[2] Ells A Hicks M Fielden M Ingram A. Severe retinopathy of prematurity: longitudinal observation of disease and screening implications. Eye (Lond). (2005) 19:138–44. 10.1038/sj.eye.6701437 15218516

[3] de Las Rivas Ramirez N Luque Aranda G Rius Diaz F Perez Frias FJ Sanchez Tamayo T. Risk factors associated with retinopathy of prematurity development and progression. Sci Rep. (2022) 12:21977. 10.1038/s41598-022-26229-4 36539470 PMC9767907

[4] Woo SJ Park KH Jung HJ Kim S Choe G Ahn J Effects of maternal and placental inflammation on retinopathy of prematurity. Graefes Arch Clin Exp Ophthalmol. (2012) 250:915–23. 10.1007/s00417-011-1648-2 21455777

[5] Manzoni P Maestri A Leonessa M Mostert M Farina D Gomirato G. Fungal and bacterial sepsis and threshold ROP in preterm very low birth weight neonates. J Perinatol. (2006) 26:23–30. 10.1038/sj.jp.7211420 16355104

[6] Lundgren P Lundberg L Hellgren G Holmstrom G Hard AL Smith LE Aggressive posterior retinopathy of prematurity is associated with multiple infectious episodes and thrombocytopenia. Neonatology. (2017) 111:79–85. 10.1159/000448161 27631399 PMC5159260

[7] Chen M Citil A McCabe F Leicht KM Fiascone J Dammann CE Infection, oxygen, and immaturity: interacting risk factors for retinopathy of prematurity. Neonatology. (2011) 99:125–32. 10.1159/000312821 20733333 PMC2939989

[8] Bonafiglia E Gusson E Longo R Ficial B Tisato MG Rossignoli S Early and late onset sepsis and retinopathy of prematurity in a cohort of preterm infants. Sci Rep. (2022) 12:11675. 10.1038/s41598-022-15804-4 35803970 PMC9270376

[9] Ali AA Gomaa NAS Awadein AR Al-Hayouti HH Hegazy AI. Retrospective cohort study shows that the risks for retinopathy of prematurity included birth age and weight, medical conditions and treatment. Acta Paediatr. (2017) 106:1919–27. 10.1111/apa.14019 28799178

[10] Gabay C Kushner I. Acute-phase proteins and other systemic responses to inflammation. N Engl J Med. (1999) 340:448–54. 10.1056/NEJM199902113400607 9971870

[11] Koch AE Polverini PJ Kunkel SL Harlow LA DiPietro LA Elner VM Interleukin-8 as a macrophage-derived mediator of angiogenesis. Science. (1992) 258:1798–801. 10.1126/science.1281554 1281554

[12] Lee J Dammann O. Perinatal infection, inflammation, and retinopathy of prematurity. Semin Fetal Neonatal Med. (2012) 17:26–9. 10.1016/j.siny.2011.08.007 21903492 PMC3242877

[13] Silveira RC Fortes Filho JB Procianoy RS. Assessment of the contribution of cytokine plasma levels to detect retinopathy of prematurity in very low birth weight infants. Invest Ophthalmol Vis Sci. (2011) 52:1297–301. 10.1167/iovs.10-6279 21071735

[14] Velez-Montoya R Clapp C Rivera JC Garcia-Aguirre G Morales-Canton V Fromow-Guerra J Intraocular and systemic levels of vascular endothelial growth factor in advanced cases of retinopathy of prematurity. Clin Ophthalmol. (2010) 4:947–53. 10.2147/OPTH.S11650 20856587 PMC2938272

[15] Pieh C Kruger M Lagreze WA Gimpel C Buschbeck C Zirrgiebel U Plasma sE-selectin in premature infants: a possible surrogate marker of retinopathy of prematurity. Invest Ophthalmol Vis Sci. (2010) 51:3709–13. 10.1167/iovs.09-4723 20181841

[16] Park YJ Woo SJ Kim YM Hong S Lee YE Park KH. Immune and inflammatory proteins in cord blood as predictive biomarkers of retinopathy of prematurity in preterm infants. Invest Ophthalmol Vis Sci. (2019) 60:3813–20. 10.1167/iovs.19-27258 31525777

[17] Woo SJ Park JY Hong S Kim YM Park YH Lee YE Inflammatory and angiogenic mediators in amniotic fluid are associated with the development of retinopathy of prematurity in preterm infants. Invest Ophthalmol Vis Sci. (2020) 61:42. 10.1167/iovs.61.5.42 32446247 PMC7405804

[18] Chiang MF Quinn GE Fielder AR Ostmo SR Paul Chan RV Berrocal A International classification of retinopathy of prematurity, third edition. Ophthalmology. (2021) 128:e51–68. 10.1016/j.ophtha.2021.05.031 34247850 PMC10979521

[19] Goldstein B Giroir B Randolph A, International Consensus Conference on Pediatric Sepsis. International pediatric sepsis consensus conference: definitions for sepsis and organ dysfunction in pediatrics. Pediatr Crit Care Med. (2005) 6:2–8. 10.1097/01.PCC.0000149131.72248.E6 15636651

[20] Tan W Li B Wang Z Zou J Jia Y Yoshida S Novel potential biomarkers for retinopathy of prematurity. Front Med (Lausanne). (2022) 9:840030. 10.3389/fmed.2022.840030 35187013 PMC8848752

[21] Ozcan B Kavurt AS Aydemir O Gencturk Z Bas AY Demirel N. SNAPPE-II and risk of neonatal morbidities in very low birth weight preterm infants. Turk J Pediatr. (2017) 59:105–12. 10.24953/turkjped.2017.02.001 29276862

[22] Fortes Filho JB Dill JC Ishizaki A Aguiar WW Silveira RC Procianoy RS. Score for neonatal acute physiology and perinatal extension II as a predictor of retinopathy of prematurity: study in 304 very-low-birth-weight preterm infants. Ophthalmologica. (2009) 223:177–82. 10.1159/000197114 19174615

[23] Dong C. Cytokine regulation and function in T cells. Annu Rev Immunol. (2021) 39:51–76. 10.1146/annurev-immunol-061020-053702 33428453

[24] Borish LC Steinke JW. 2. Cytokines and chemokines. J Allergy Clin Immunol. (2003) 111:S460–75. 10.1067/mai.2003.108 12592293

[25] Sood BG Madan A Saha S Schendel D Thorsen P Skogstrand K Perinatal systemic inflammatory response syndrome and retinopathy of prematurity. Pediatr Res. (2010) 67:394–400. 10.1203/PDR.0b013e3181d01a36 20032809 PMC2873779

[26] Roberts AW. G-CSF: a key regulator of neutrophil production, but that’s not all!. Growth Factors. (2005) 23:33–41. 10.1080/08977190500055836 16019425

[27] Minamino K Adachi Y Okigaki M Ito H Togawa Y Fujita K Macrophage colony-stimulating factor (M-CSF), as well as granulocyte colony-stimulating factor (G-CSF), accelerates neovascularization. Stem Cells. (2005) 23:347–54. 10.1634/stemcells.2004-0190 15749929

[28] Podar K Anderson KC. The pathophysiologic role of VEGF in hematologic malignancies: therapeutic implications. Blood. (2005) 105:1383–95. 10.1182/blood-2004-07-2909 15471951

[29] Capoluongo E Vento G Ameglio F Lulli P Matassa PG Carrozza C Increased levels of IGF-1 and beta2-microglobulin in epithelial lining fluid of preterm newborns developing chronic lung disease. Effects of rhG-CSF. Int J Immunopathol Pharmacol. (2006) 19:57–66. 10.1177/205873920601900106 16569340

[30] Sato T Kusaka S Shimojo H Fujikado T. Simultaneous analyses of vitreous levels of 27 cytokines in eyes with retinopathy of prematurity. Ophthalmology. (2009) 116:2165–9. 10.1016/j.ophtha.2009.04.026 19700197

[31] Lotfi N Thome R Rezaei N Zhang GX Rezaei A Rostami A Roles of GM-CSF in the pathogenesis of autoimmune diseases: an update. Front Immunol. (2019) 10:1265. 10.3389/fimmu.2019.01265 31275302 PMC6593264

[32] Yang S Wang J Brand DD Zheng SG. Role of TNF-TNF receptor 2 signal in regulatory T cells and its therapeutic implications. Front Immunol. (2018) 9:784. 10.3389/fimmu.2018.00784 29725328 PMC5916970

[33] Wan T Xu Z Zhou HJ Zhang H Luo Y Li Y Functional analyses of TNFR2 in physiological and pathological retina angiogenesis. Invest Ophthalmol Vis Sci. (2013) 54:211–21. 10.1167/iovs.12-10364 23188724 PMC3544528

[34] Cooper AM Khader SA. IL-12p40: an inherently agonistic cytokine. Trends Immunol. (2007) 28:33–8. 10.1016/j.it.2006.11.002 17126601

[35] Zhumalina AK Tusupkaliev BT Zharlykasinova MB Zhekeyeva BA Darzhanova KB. The levels of pro- and anti-inflammatory cytokines in premature infants with perinatal infections. Mol Cell Biochem. (2022) 477:621–5. 10.1007/s11010-021-04314-y 34860348

[36] Kany S Vollrath JT Relja B. Cytokines in inflammatory disease. Int J Mol Sci. (2019) 20(23):6008. 10.3390/ijms20236008 31795299 PMC6929211

[37] Dougan M Dranoff G Dougan SK. GM-CSF, IL-3, and IL-5 family of cytokines: regulators of inflammation. Immunity. (2019) 50:796–811. 10.1016/j.immuni.2019.03.022 30995500 PMC12512237

[38] Lin YW Chen SN Muo CH Sung FC Lin MH. Risk of retinopathy of prematurity in preterm births with respiratory distress syndrome: a population-based cohort study in Taiwan. Int J Gen Med. (2022) 15:2149–62. 10.2147/IJGM.S344056 35241930 PMC8887609

[39] Chang JW. Risk factor analysis for the development and progression of retinopathy of prematurity. PLoS One. (2019) 14:e0219934. 10.3390/ijms20236008 31318921 PMC6638955

[40] Dong Y Fischer R Naude PJ Maier O Nyakas C Duffey M Essential protective role of tumor necrosis factor receptor 2 in neurodegeneration. Proc Natl Acad Sci U S A. (2016) 113:12304–9. 10.1073/pnas.1605195113 27791020 PMC5087045

[41] Papazian I Tsoukala E Boutou A Karamita M Kambas K Iliopoulou L Fundamentally different roles of neuronal TNF receptors in CNS pathology: tNFR1 and IKKbeta promote microglial responses and tissue injury in demyelination while TNFR2 protects against excitotoxicity in mice. J Neuroinflammation. (2021) 18:222. 10.1186/s12974-021-02200-4 34565380 PMC8466720

[42] Wiesolek HL Bui TM Lee JJ Dalal P Finkielsztein A Batra A Intercellular adhesion molecule 1 functions as an efferocytosis receptor in inflammatory macrophages. Am J Pathol. (2020) 190:874–85. 10.1016/j.ajpath.2019.12.006 32035057 PMC7180595

[43] Yu KY Yung S Chau MK Tang CS Yap DY Tang AH Clinico-pathological associations of serum VCAM-1 and ICAM-1 levels in patients with lupus nephritis. Lupus. (2021) 30:1039–50. 10.1177/09612033211004727 33765901

[44] Baregamian N Song J Bailey CE Papaconstantinou J Evers BM Chung DH. Tumor necrosis factor-alpha and apoptosis signal-regulating kinase 1 control reactive oxygen species release, mitochondrial autophagy, and c-jun N-terminal kinase/p38 phosphorylation during necrotizing enterocolitis. Oxid Med Cell Longev. (2009) 2:297–306. 10.4161/oxim.2.5.9541 20716917 PMC2835918

[45] Tremblay E Ferretti E Babakissa C Burghardt KM Levy E Beaulieu JF. IL-17-related signature genes linked to human necrotizing enterocolitis. BMC Res Notes. (2021) 14:82. 10.1186/s13104-021-05489-9 33663574 PMC7934396

[46] Tremblay E Thibault MP Ferretti E Babakissa C Bertelle V Bettolli M Gene expression profiling in necrotizing enterocolitis reveals pathways common to those reported in crohn’s disease. BMC Med Genomics. (2016) 9:6. 10.1186/s12920-016-0166-9 26801768 PMC4722613

[47] Cutler P. Protein arrays: the current state-of-the-art. Proteomics. (2003) 3:3–18. 10.1002/pmic.200390007 12548629

[48] Liu BM Martins TB Peterson LK Hill HR. Clinical significance of measuring serum cytokine levels as inflammatory biomarkers in adult and pediatric COVID-19 cases: a review. Cytokine. (2021) 142:155478. 10.1016/j.cyto.2021.155478 33667962 PMC7901304

