# Supplementary material for: Association of serum levels of inflammatory cytokines with retinopathy of prematurity in preterm infants
Source: Front Pediatr. 2024 Jan 8;11:1195904. doi: 10.3389/fped.2023.1195904 (PMC10800500; doi:10.3389/fped.2023.1195904)
Supplement: Supplementary file 1 [file Table1.docx]

| **Supplementary Table 1.**  **ROP Characteristics of Premature Infants (N=50)** | | | | |
| --- | --- | --- | --- | --- |
| Variables | No ROP (n=14) | Mild ROP (n=14) | Type 1 ROP (n=22) | P values |
| **ROP Characteristics** |  |  |  |  |
| **Zone** |  |  |  | P = 0.001* |
| Zone 1, n (%) | 0 (0.0)^[A]^ | 0 (0.0)^[B]^ | 4 (18.2)^[C]^ |  |
| Zone 2, n (%) | 0 (0.0)^[A]^ | 13 (92.9)^[B]^ | 18 (81.8)^[B]^ |  |
| Zone 3, n (%) | 0 (0.0)^[A]^ | 1 (7.1)^[B]^ | 0 (0.0)^[B]^ |  |
| **Stage** |  |  |  | P < 0.05* |
| Stage 1, n (%) | 0 (0.0)^[A]^ | 5 (35.7)^[B]^ | 0 (0.0)^[C]^ |  |
| Stage 2, n (%) | 0 (0.0)^[A]^ | 7 (50.0)^[B]^ | 1 (4.5)^[C]^ |  |
| Stage 3, n (%) | 0 (0.0)^[A]^ | 2 (14.3)^[B]^ | 21 (95.5)^[C]^ |  |
| Plus disease, n (%) | 0 (0.0)^[A]^ | 0 (0.0)^[A]^ | 20 (90.0)^[B]^ | P < 0.05* |

Abbreviations: ROP: Retinopathy of Prematurity;
[A, B, C]: Inter-group comparisons using post hoc test; different (or the same) letters represent significant (or insignificant) statistical differences.
