# Supplementary material for: Association of serum levels of inflammatory cytokines with retinopathy of prematurity in preterm infants
Source: Front Pediatr. 2024 Jan 8;11:1195904. doi: 10.3389/fped.2023.1195904 (PMC10800500; doi:10.3389/fped.2023.1195904)
Supplement: Supplementary file 2 [file Table2.docx]

| **Supplementary Table 2. Serum Inflammatory Cytokines Changes from Baseline up to 4 weeks after IVI treatment (N = 22)** | | | | | | |
| --- | --- | --- | --- | --- | --- | --- |
| **Time** | **N** | **Median (Range), pg/mL** | | **Mean ± SD, pg/mL** | | **P value** |
| **BLC** |  |  |  |  |  |  |
| Baseline | 22 | 9.97 (5.20–15.10) | | 11.54 ± 7.60 | | N/A |
| 2 weeks | 22 | 9.75 (4.78 – 14.61) | | 11.13 ± 7.05 | | 0.4392 |
| 4 weeks | 22 | 8.99 (7.24 – 14.78) | | 12.39 ± 9.90 | | 0.4780 |
|  |  |  | |  | |  |
| **Eotaxin** |  |  |  |  |  |  |
| Baseline | 22 | 292.83 (192.98 – 472.24) | | 308.27 ± 165.81 | | N/A |
| 2 weeks | 22 | 325.67 (176.41 – 468.90) | | 351.71 ± 225.25 | | 0.3336 |
| 4 weeks | 22 | 332.07 (140.96 – 443.94) | | 337.27 ± 209.28 | | 0.3500 |
|  |  |  | |  | |  |
| **Eotaxin2** |  |  |  |  |  |  |
| Baseline | 22 | 262.74 (172.02 – 356.27) | | 259.91 ± 136.82 | | N/A |
| 2 weeks | 22 | 268.18 (231.12 – 426.43) | | 313.65 ± 181.65 | | 0.1838 |
| 4 weeks | 22 | 262.28 (165.24 – 411.33) | | 313.67 ± 184.24 | | 0.3669 |
|  |  |  | |  | |  |
| **GCSF** |  |  |  |  | |  |
| Baseline | 22 | 19.05 (4.85 – 49.40) | | 109.79 ± 267.12 | | N/A |
| 2 weeks | 22 | 8.23 (4.00 – 22.72) | | 13.36 ± 13.32 | | **0.0035*** |
| 4 weeks | 22 | 11.58 (3.07 – 22.20) | | 172.40 ± 709.47 | | 0.1756 |
|  |  |  | |  | |  |
| **GMCSF** |  |  |  |  |  |  |
| Baseline | 22 | 50.82 (0.00 – 80.44) | | 52.79 ± 48.01 | | N/A |
| 2 weeks | 22 | 63.23 (10.22 – 104.45) | | 76.35 ± 79.15 | | 0.2413 |
| 4 weeks | 22 | 87.18 (39.94 – 116.00) | | 125.73 ± 141.23 | | **0.0425*** |
|  |  |  | |  | |  |
| **I309** |  |  |  |  |  |  |
| Baseline | 22 | 0.00 (0.00 – 11.47) | | 10.47 ± 23.85 | | N/A |
| 2 weeks | 22 | 0.00 (0.00 – 1.57) | | 5.13 ± 11.95 | | 0.4973 |
| 4 weeks | 22 | 0.01 (0.00 – 3.91) | | 51.12 ± 187.76 | | 0.6257 |
|  |  |  | |  | |  |
| **ICAM** |  |  |  |  |  |  |
| Baseline | 22 | 3340.34 (2881.95 – 3682.14) | | 3302.98 ± 470.72 | | N/A |
| 2 weeks | 22 | 3316.03 (2878.72 – 3671.71) | | 3234.94 ± 622.94 | | 0.3843 |
| 4 weeks | 22 | 3308.47 (2795.24 – 3557.26) | | 3133.87 ± 629.35 | | **0.0467*** |
|  |  |  | |  | |  |
| **IFN-1a** |  |  |  |  |  |  |
| Baseline | 22 | 0.00 (0.00 – 1.01) | | 7.13 ± 17.02 | | N/A |
| 2 weeks | 22 | 0.00 (0.00 – 2.18) | | 5.64 ± 13.92 | | 1.000 |
| 4 weeks | 22 | 0.00 (0.00 – 3.13) | | 6.60 ± 17.56 | | 0.7695 |
|  |  |  | |  | |  |
| **IFN-1b** |  |  |  |  |  |  |
| Baseline | 22 | 1.16 (0.00 – 6.58) | | 4.44 ± 6.53 | | N/A |
| 2 weeks | 22 | 0.75 (0.00 – 5.19) | | 2.83 ± 4.01 | | 0.2435 |
| 4 weeks | 22 | 1.41 (0.00 – 4.01) | | 5.86 ± 12.03 | | 0.7987 |
|  |  |  | |  | |  |
| **IFNg** |  |  |  |  |  |  |
| Baseline | 22 | 3.48 (1.55 – 5.19) | | 5.65 ± 9.96 | | N/A |
| 2 weeks | 22 | 1.98 (1.07 – 5.91) | | 5.67 ± 9.86 | | 0.0650 |
| 4 weeks | 22 | 2.75 (0.95 – 11.58) | | 25.19 ± 70.41 | | 0.8631 |
|  |  |  | |  | |  |
| **IL10** |  |  | |  | |  |
| Baseline | 22 | 32.24 (13.33 – 61.08) | | 39.72 ± 34.51 | | N/A |
| 2 weeks | 22 | 22.36 (8.27 – 34.98) | | 31.29 ± 37.19 | | 0.2190 |
| 4 weeks | 22 | 24.10 (16.06 – 50.48) | | 41.96 ± 43.41 | | 0.5392 |
|  |  |  | |  | |  |
| **IL11** |  |  | |  | |  |
| Baseline | 22 | 130.28 (9.44 – 221. 8) | | 151.52 ± 161.46 | | N/A |
| 2 weeks | 22 | 63.29 (26.57 – 223.42) | | 128.62 ± 165.51 | | 0.5113 |
| 4 weeks | 22 | 80.79 (14.53 – 251.02) | | 174.86 ± 204.91 | | 0.6794 |
|  |  |  | |  | |  |
| **IL12p40** |  |  | |  | |  |
| Baseline | 22 | 14.97 (7.26 – 20.25) | | 15.82 ± 10.99 | | N/A |
| 2 weeks | 22 | 12.79 (4.69 – 17.68) | | 13.16 ± 10.20 | | 0.1951 |
| 4 weeks | 22 | 15.27 (7.46 – 22.98) | | 41.25 ± 110.22 | | 0.1342 |
|  |  |  | |  | |  |
| **IL12p70** |  |  | |  | |  |
| Baseline | 22 | 0.29 (0.00 – 0.86) | | 0.51 ± 0.62 | | N/A |
| 2 weeks | 22 | 0.16 (00 – 0.52) | | 0.36 ± 0.45 | | 0.4171 |
| 4 weeks | 22 | 0.25 (0.09 - .62) | | 0.62 ± 0.73 | | 0.8983 |
|  |  |  | |  | |  |
| **IL13** |  |  | |  | |  |
| Baseline | 22 | 1.03 (0.70 – 1.71) | | 1.29 ± 0.99 | | N/A |
| 2 weeks | 22 | 0.99 (0.45 – 2.04) | | 1.31 ± 1.11 | | 0.8801 |
| 4 weeks | 22 | 1.17 (0.64 – 3.61) | | 2.26 ± 2.80 | | 0.4477 |
|  |  |  | |  | |  |
| **IL15** |  |  | |  | |  |
| Baseline | 22 | 1.43 (0.69 – 4.00) | | 4.31 ± 10.37 | | N/A |
| 2 weeks | 22 | 1.58 ( 0.73 – 4.23) | | 4.76 ± 10.53 | | 0.9599 |
| 4 weeks | 22 | 2.08 (0.66 – 4.53) | | 8.15 ± 14.14 | | 0.9625 |
|  |  |  | |  | |  |
| **IL16** |  |  | |  | |  |
| Baseline | 22 | 95.08 (48.82 – 189.92) | | 188.38 ± 256.08 | | N/A |
| 2 weeks | 22 | 71.63 (43.50 – 226.17) | | 181.25 ± 239.19 | | 0.8878 |
| 4 weeks | 22 | 99.37 (52.55 – 234.38) | | 180.04 ± 247.61 | | 0.9375 |
|  |  |  | |  | |  |
| **IL17** |  |  | |  | |  |
| Baseline | 22 | 5.40 (2.33 – 12.27) | | 8.23 ± 8.71 | | N/A |
| 2 weeks | 22 | 5.25 (2.42 – 10.78) | | 6.86 ± 7.04 | | 0.4304 |
| 4 weeks | 22 | 7.30 (1.93 – 17.60) | | 13.26 ± 16.98 | | 0.3736 |
|  |  |  | |  | |  |
| **IL1ra** |  |  | |  | |  |
| Baseline | 22 | 2.71 (0.00 – 5.80) | | 4.59 ± 6.16 | | N/A |
| 2 weeks | 22 | 2.79 (0.00 – 4.97) | | 7.09 ± 17.24 | | 0.7436 |
| 4 weeks | 22 | 3.06 (0.00 – 5.67) | | 32.46 ± 132.07 | | 0.5477 |
|  |  |  | |  | |  |
| **IL2** |  |  | |  | |  |
| Baseline | 22 | 25.04 (9.76 – 31.04) | | 23.60 ± 20.06 | | N/A |
| 2 weeks | 22 | 24.65 (9.52 – 30.90) | | 26.32 ± 22.30 | | 0.6023 |
| 4 weeks | 22 | 21.54 (15.49 – 46.69) | | 36.36 ± 34.85 | | 0.0760 |
|  |  |  | |  | |  |
| **IL4** |  |  | |  | |  |
| Baseline | 22 | 4.36 (0.00 -15.27) | | 11.56 ± 16.94 | | N/A |
| 2 weeks | 22 | 6.02 (0.00 – 18.61) | | 10.01 ± 12.78 | | 0.3692 |
| 4 weeks | 22 | 10.60 (1.84 – 20.33) | | 17.37 ± 26.31 | | 0.4091 |
|  |  |  | |  | |  |
| **IL5** |  |  | |  | |  |
| Baseline | 22 | 12.23 (7.94 – 21.93) | | 17.37 ± 15.36 | | N/A |
| 2 weeks | 22 | 13.48 (6.55 – 24.05) | | 16.19 ± 11.76 | | 0.7897 |
| 4 weeks | 22 | 16.73 (11.99 – 45.26) | | 28.14 ± 28.51 | | 0.2870 |
|  |  |  | |  | |  |
| **IL6** |  |  | |  | |  |
| Baseline | 22 | 9.14 (4.13 – 16.50) | | 67.72 ± 258.98 | | N/A |
| 2 weeks | 22 | 7.01 (3.76 – 13.11) | | 12.05 ± 14.66 | | 0.3336 |
| 4 weeks | 22 | 10.40 (6.15 – 37.07) | | 141.70 ± 504.85 | | 0.4205 |
|  |  |  | |  | |  |
| **IL6R** |  |  | |  | |  |
| Baseline | 22 | 4688.76 (4385.96 – 5142.75) | | 4668.23 ± 607.39 | | N/A |
| 2 weeks | 22 | 4767.68 (4435.27 – 5079.71) | | 4730 ± 792.19 | | 0.4205 |
| 4 weeks | 22 | 4808.31 (4204.96 – 5034.67) | | 4408.21 ± 1095.51 | | 0.3176 |
|  |  |  | |  | |  |
| **IL7** |  |  | |  | |  |
| Baseline | 22 | 56.97 (21.11 – 117.74) | | 71.05 ± 58.18 | | N/A |
| 2 weeks | 22 | 65.18 (28.12 – 111.09) | | 78.44 ± 65.22 | | 0.4780 |
| 4 weeks | 22 | 93.62 (56.29 – 132.04) | | 146.43 ± 184.29 | | 0.1095 |
|  |  |  | |  | |  |
| **IL8** |  |  | |  | |  |
| Baseline | 22 | 12.50 (2.47 – 34.17) | | 19.80 ± 22.71 | | N/A |
| 2 weeks | 22 | 10.59 (4.62 – 17.15) | | 15.12 ± 21.24 | | 0.1528 |
| 4 weeks | 22 | 6.89 (3.55 -30.87) | | 33.91 ± 75.05 | | 0.4980 |
|  |  |  | |  | |  |
| **MCP1** |  |  | |  | |  |
| Baseline | 22 | 272.98 (180.95 – 371.58) | | 289.56 ± 139.74 | | N/A |
| 2 weeks | 22 | 249.01 (164.29 – 300.73) | | 241.17 ± 99.90 | | 0.1627 |
| 4 weeks | 22 | 212.03 (163.39 – 284.43) | | 235.79 ± 123.55 | | 0.0820 |
|  |  |  | |  | |  |
| **MCSF** |  |  | |  | |  |
| Baseline | 22 | 0.03 (0.00 – 1.80) | | 1.21 ± 1.90 | | N/A |
| 2 weeks | 22 | 0.10 (0.00 – 0.75) | | 0.62 ± 1.19 | | 0.1465 |
| 4 weeks | 22 | 0.24 (0.00 – 0.86) | | 2.50 ± 8.09 | | 0.4543 |
|  |  |  | |  | |  |
| **MIG** |  |  | |  | |  |
| Baseline | 22 | 76.81 (18.40 – 148.55) | | 119.48 ± 161.61 | | N/A |
| 2 weeks | 22 | 38.59 (23.09 – 113.88) | | 79.40 ± 87.02 | | 0.2688 |
| 4 weeks | 22 | 60.72 (21.28 – 122.60) | | 419.49 ± 1499.46 | | 0.9066 |
|  |  |  | |  | |  |
| **M1P1a** |  |  | |  | |  |
| Baseline | 22 | 85.19 (45.73 – 153.88) | | 120.40 ± 110.47 | | N/A |
| 2 weeks | 22 | 86.62 (53.15 – 101.02) | | 96.64 ± 74.46 | | 0.5604 |
| 4 weeks | 22 | 84.96 (53.37 – 126.67) | | 144.78 ± 216.89 | | 0.6947 |
|  |  |  | |  | |  |
| **M1P1b** |  |  | |  | |  |
| Baseline | 22 | 24.85 (17.25 – 61.58) | | 38.49 ± 29.53 | | N/A |
| 2 weeks | 22 | 34.88 (15.89 – 45.79) | | 35.39 ± 22.85 | | 0.7897 |
| 4 weeks | 22 | 31.59 (21.70 – 51.64) | | 43.24 ± 46.21 | | 0.7181 |
|  |  |  | |  | |  |
| **M1P1d** |  |  | |  | |  |
| Baseline | 22 | 274.03 (236.69 – 347.65) | | 289.13 ± 72.79 | | N/A |
| 2 weeks | 22 | 276.54 (260.47 – 335.13) | | 290.71 ± 65.93 | | 0.6715 |
| 4 weeks | 22 | 292.33 (249.94 – 339.86) | | 293.17 ± 71.18 | | 0.8140 |
|  |  |  | |  | |  |
| **PDGFBB** |  |  | |  | |  |
| Baseline | 22 | 16097.83 (10707.49 – 20212.70) | | 14779.41 ± 5714.37 | | N/A |
| 2 weeks | 22 | 16579.56 (12213.35 – 20522.66) | | 16226.82 ± 5296.02 | | 0.3176 |
| 4 weeks | 22 | 17089.76 (13642.99 – 20945.29) | | 17556.27 ± 5068.92 | | 0.0703 |
|  |  |  | |  | |  |
| **RANTES** |  |  | |  | |  |
| Baseline | 22 | 5767.36 (4795.31 – 6545.69) | | 5642.81 ± 1011.73 | | N/A |
| 2 weeks | 22 | 5795.36 (5381.62 – 6408.22) | | 5680.74 ± 925.50 | | 0.7181 |
| 4 weeks | 22 | 5855.73 (4901.99 – 6064.08) | | 5581.67 ± 970.45 | | 0.5392 |
|  |  |  | |  | |  |
| **TIMP1** |  |  | |  | |  |
| Baseline | 22 | 4004.38 (3838.04 – 4298.02) | | 4061.44 ± 455.81 | | N/A |
| 2 weeks | 22 | 4104.81 (3821.27 – 4334.16) | | 4101.28 ± 387.97 | | 0.5184 |
| 4 weeks | 22 | 3981.35 (3758.78 – 4166.84) | | 3937.92 ± 474.44 | | 0.1433 |
|  |  |  | |  | |  |
| **TIMP2** |  |  | |  | |  |
| Baseline | 22 | 4898.23 (4170.42 – 5821.37) | | 4790.48 ± 1284.50 | | N/A |
| 2 weeks | 22 | 5311.66 (4124.53 – 5773.04) | | 4939.41 ± 1275.48 | | 0.9126 |
| 4 weeks | 22 | 5154.82 (4187.49 – 5762.94) | | 4886.08 ± 1349.91 | | 0.5184 |
|  |  |  | |  | |  |
| **TNFR1** |  |  | |  | |  |
| Baseline | 22 | 7079.50 (6363.80 – 7875.74) | | 7188.76 ± 1446.89 | | N/A |
| 2 weeks | 22 | 6735.79 (6035.16 – 7448.03) | | 6865.99 ± 1559.78 | | 0.0553 |
| 4 weeks | 22 | 6815.12 (5618.24 – 7777.82) | | 6777.90 ± 1805.05 | | 0.0703 |
|  |  |  | |  | |  |
| **TNFα** |  |  | |  | |  |
| Baseline | 22 | 3.96 (1.29 – 6.57) | | 5.06 ± 5.31 | | N/A |
| 2 weeks | 22 | 4.25 (1.45 – 7.00) | | 4.62 ± 3.50 | | 0.6261 |
| 4 weeks | 22 | 3.73 (2.53 – 7.24) | | 8.15 ± 14.61 | | 0.9375 |
|  |  |  | |  | |  |
| **TNFβ** |  |  | |  | |  |
| Baseline | 22 | 413.34 (148.04 – 703.12) | | 508.01 ± 550.17 | | N/A |
| 2 weeks | 22 | 375.18 (157.68 – 704.40) | | 515.58 ± 422.27 | | 0.8801 |
| 4 weeks | 22 | 416.58 (228.60 – 894.19) | | 706.06 ± 667.40 | | 0.3021 |
|  |  |  | |  | |  |
| **TNFR2** |  |  |  |  |  |  |
| Baseline | 22 | 7383.83 (6771.80 – 8371.63) | | 7421.49 ± 1431.00 | | N/A |
| 2 weeks | 22 | 6862.43 (6038.01 – 7897.22) | | 6912.07 ± 1805.74 | | **0.0246*** |
| 4 weeks | 22 | 6524.56 (5412.89 – 7829.56) | | 6582.40 ± 2200.12 | | **0.0467*** |
|  |  |  | |  | |  |
| P value was evaluated by Wilcoxon signed-rank test (compared to baseline);  * Significance shown at p<0.05 | | | | | | |
