# Supplementary material for: Association of serum levels of inflammatory cytokines with retinopathy of prematurity in preterm infants
Source: Front Pediatr. 2024 Jan 8;11:1195904. doi: 10.3389/fped.2023.1195904 (PMC10800500; doi:10.3389/fped.2023.1195904)
Supplement: Supplementary file 3 [file Table3.docx]

| **Supplementary Table 3. Serum Inflammatory Cytokines Changes from Baseline up to 4 Weeks in Mild ROP (N = 14)** | | | | | | |
| --- | --- | --- | --- | --- | --- | --- |
| **Time** | **N** | **Median (Range), pg/mL** | | **Mean ± SD, pg/mL** | | **P value** |
| **BLC** |  |  |  |  |  |  |
| Baseline | 14 | 7.59 (4.88 – 13.39) | | 9.54 ± 6.31 | | N/A |
| 2 weeks | 14 | 8.56 (6.28 – 10.30) | | 8.70 ± 4.15 | | 0.5416 |
| 4 weeks | 14 | 9.67 (6.43 – 11.27) | | 9.11 ± 4.14 | | 0.7148 |
|  |  |  | |  | |  |
| **Eotaxin** |  |  |  |  |  |  |
| Baseline | 14 | 373.49 (199.86 – 507.10) | | 410.73 ± 267.37 | | N/A |
| 2 weeks | 14 | 326.11 (255.91 – 471.74) | | 370.08 ± 159.52 | | 0.3910 |
| 4 weeks | 14 | 331.05 (247.29 – 413.66) | | 338.12 ± 103.99 | | 0.4631 |
|  |  |  | |  | |  |
| **Eotaxin2** |  |  |  |  |  |  |
| Baseline | 14 | 331.67 (203.75 – 403.51) | | 316.89 ± 166.38 | | N/A |
| 2 weeks | 14 | 337.85 (192.39 – 387.44) | | 301.34 ± 123.36 | | 0.6698 |
| 4 weeks | 14 | 278.89 (212.79 – 348.89) | | 288.21 ± 110.10 | | 0.6257 |
|  |  |  | |  | |  |
| **GCSF** |  |  |  |  | |  |
| Baseline | 14 | 21.15 (8.48 – 29.53) | | 18.86 ± 13.43 | | N/A |
| 2 weeks | 14 | 16.20 (7.80 – 25.91) | | 17.92 ± 14.65 | | 0.9515 |
| 4 weeks | 14 | 10.77 (7.31 – 20.29) | | 20.04 ± 22.80 | | 0.5416 |
|  |  |  | |  | |  |
| **GMCSF** |  |  |  |  |  |  |
| Baseline | 14 | 72.10 (41.09 – 100.47) | | 73.24 ± 40.21 | | N/A |
| 2 weeks | 14 | 51.39 (30.64 – 111.36) | | 77.53 ± 64.15 | | 0.9515 |
| 4 weeks | 14 | 89.54 (54.92 – 119.84) | | 84.27 ± 46.92 | | 0.4631 |
|  |  |  | |  | |  |
| **I309** |  |  |  |  |  |  |
| Baseline | 14 | 0.42 (0.00 – 13.61) | | 7.98 ± 13.07 | | N/A |
| 2 weeks | 14 | 0.00 (0.00 – 5.95) | | 5.06 ± 10.21 | | 0.5469 |
| 4 weeks | 14 | 0.00 (0.00 – 29.26) | | 32.50 ± 85.35 | | 0.6523 |
|  |  |  | |  | |  |
| **ICAM** |  |  |  |  |  |  |
| Baseline | 14 | 3183.31 (2841.07 – 3580.39) | | 3141.41 ± 469.53 | | N/A |
| 2 weeks | 14 | 3146.19 (2878.19 – 3375.04) | | 3129.90 ± 420.52 | | 0.8552 |
| 4 weeks | 14 | 2947.40 (2511.12 – 3328.47) | | 2925.59 ± 498.85 | | 0.0906 |
|  |  |  | |  | |  |
| **IF1a** |  |  |  |  |  |  |
| Baseline | 14 | 0.00 (0.00 – 2.19) | | 0.99 ± 1.69 | | N/A |
| 2 weeks | 14 | 0.00 (0.00 – 0.00) | | 2.27 ± 8.21 | | 0.4375 |
| 4 weeks | 14 | 0.00 (0.00 – 2.59) | | 2.12 ± 3.73 | | 0.4258 |
|  |  |  | |  | |  |
| **IF1b** |  |  |  |  |  |  |
| Baseline | 14 | 1.87 (0.00 – 4.78) | | 2.39 ± 2.50 | | N/A |
| 2 weeks | 14 | 1.24 (0.00 – 2.82) | | 2.15 ± 2.88 | | 0.6377 |
| 4 weeks | 14 | 2.24 (0.00 – 4.99) | | 2.88 ± 2.79 | | 0.9460 |
|  |  |  | |  | |  |
| **IFNg** |  |  |  |  |  |  |
| Baseline | 14 | 1.42 (0.20 – 2.43) | | 1.99 ± 2.17 | | N/A |
| 2 weeks | 14 | 0.64 (0.00 – 1.82) | | 8.17 ± 21.32 | | 0.7910 |
| 4 weeks | 14 | 2.06 (0.14 – 3.78) | | 11.17 ± 23.97 | | 0.2163 |
|  |  |  | |  | |  |
| **IL10** |  |  | |  | |  |
| Baseline | 14 | 25.74 (17.93 – 36.67) | | 27.58 ± 16.56 | | N/A |
| 2 weeks | 14 | 23.91 (14.17 – 33.14) | | 22.94 ± 11.65 | | 0.3258 |
| 4 weeks | 14 | 30.33 (19.53 – 42.17) | | 28.39 ± 15.14 | | 0.9032 |
|  |  |  | |  | |  |
| **IL11** |  |  | |  | |  |
| Baseline | 14 | 129.26 (30.85 – 289.26) | | 157.71 ± 123.24 | | N/A |
| 2 weeks | 14 | 90.41 (1.92 – 245.30) | | 161.41 ± 204.10 | | 0.4143 |
| 4 weeks | 14 | 101.13 (17.82 – 272.87) | | 145.78 ± 133.99 | | 0.6772 |
|  |  |  | |  | |  |
| **IL12p40** |  |  | |  | |  |
| Baseline | 14 | 9.05 (4.79 – 18.19) | | 14.65 ± 15.43 | | N/A |
| 2 weeks | 14 | 17.33 (10.54 – 25.20) | | 18.04 ± 11.08 | | 0.2676 |
| 4 weeks | 14 | 17.31 (7.93 – 27.26) | | 19.32 ± 12.20 | | 0.3258 |
|  |  |  | |  | |  |
| **IL12p70** |  |  | |  | |  |
| Baseline | 14 | 0.47 (0.20 – 0.77) | | 0.48 ± 0.40 | | N/A |
| 2 weeks | 14 | 0.34 (0.00 – 0.64) | | 0.46 ± 0.56 | | 0.5186 |
| 4 weeks | 14 | 0.46 (0.25 – 0.74) | | 0.50 ± 0.38 | | 0.7148 |
|  |  |  | |  | |  |
| **IL13** |  |  | |  | |  |
| Baseline | 14 | 0.89 (0.61 – 1.36) | | 1.00 ± 0.57 | | N/A |
| 2 weeks | 14 | 0.86 (0.26 – 1.80) | | 1.32 ± 1.64 | | 1.0000 |
| 4 weeks | 14 | 1.15 (0.50 – 2.43) | | 1.70 ± 1.86 | | 0.5830 |
|  |  |  | |  | |  |
| **IL15** |  |  | |  | |  |
| Baseline | 14 | 1.28 (0.62 – 2.16) | | 1.48 ± 1.21 | | N/A |
| 2 weeks | 14 | 0.62 (0.25 – 0.87) | | 4.95 ± 13.45 | | 0.8926 |
| 4 weeks | 14 | 1.48 (0.67 – 3.41) | | 8.24 ± 17.50 | | 0.3258 |
|  |  |  | |  | |  |
| **IL16** |  |  | |  | |  |
| Baseline | 14 | 549.65 (220.65 – 863.94) | | 568.29 ± 361.36 | | N/A |
| 2 weeks | 14 | 449.28 (179.95 – 637.45) | | 467.12 ± 320.55 | | 0.5016 |
| 4 weeks | 14 | 420.23 (313.96 – 826.20) | | 513.10 ± 318.29 | | 0.8552 |
|  |  |  | |  | |  |
| **IL17** |  |  | |  | |  |
| Baseline | 14 | 4.14 (0.00 – 8.63) | | 4.95 ± 4.91 | | N/A |
| 2 weeks | 14 | 2.97 (0.00 – 7.46) | | 6.92 ± 12.30 | | 0.7002 |
| 4 weeks | 14 | 3.74 (0.00 – 9.77) | | 6.31 ± 6.51 | | 0.3013 |
|  |  |  | |  | |  |
| **IL1ra** |  |  | |  | |  |
| Baseline | 14 | 1.46 (0.00 – 4.45) | | 2.19 ± 2.36 | | N/A |
| 2 weeks | 14 | 1.31 (0.00 – 3.21) | | 1.59 ± 1.66 | | 0.3125 |
| 4 weeks | 14 | 1.67 (0.14 – 4.25) | | 2.89 ± 3.25 | | 0.7646 |
|  |  |  | |  | |  |
| **IL2** |  |  | |  | |  |
| Baseline | 14 | 24.15 (18.45 – 37.28) | | 26.00 ± 16.28 | | N/A |
| 2 weeks | 14 | 19.29 (15.84 – 28.29) | | 19.75 ± 11.26 | | 0.2166 |
| 4 weeks | 14 | 21.45 (15.01 – 26.62) | | 21.20 ± 11.01 | | 0.6257 |
|  |  |  | |  | |  |
| **IL4** |  |  | |  | |  |
| Baseline | 14 | 2.67 (0.00 – 8.00) | | 4.42 ± 4.65 | | N/A |
| 2 weeks | 14 | 2.41 (0.00 -6.36) | | 8.80 ± 19.57 | | 0.9219 |
| 4 weeks | 14 | 10.47 (5.34 – 13.13) | | 9.53 ± 7.43 | | 0.0640 |
|  |  |  | |  | |  |
| **IL5** |  |  | |  | |  |
| Baseline | 14 | 12.35 (8.69 – 17.34) | | 13.71 ± 8.85 | | N/A |
| 2 weeks | 14 | 10.63 (3.24 – 15.48) | | 14.22 ± 18.28 | | 0.5830 |
| 4 weeks | 14 | 14.05 (8.59 – 25.78) | | 20.89 ± 25.10 | | 0.6257 |
|  |  |  | |  | |  |
| **IL6** |  |  | |  | |  |
| Baseline | 14 | 10.17 (5.07 – 12.36) | | 9.98 ± 6.08 | | N/A |
| 2 weeks | 14 | 7.20 (5.35 – 9.85) | | 17.02 ± 37.83 | | 0.5016 |
| 4 weeks | 14 | 9.52 (4.53 – 10.68) | | 24.04 ± 57.06 | | 0.8077 |
|  |  |  | |  | |  |
| **IL6R** |  |  | |  | |  |
| Baseline | 14 | 5339.49 (5115.76 – 5541.36) | | 5264.96 ± 484.23 | | N/A |
| 2 weeks | 14 | 5257.72 (4876.30 – 5550.76) | | 5125.28 ± 447.13 | | 0.6257 |
| 4 weeks | 14 | 5178.08 (4906.41 – 5440.36) | | 5072.61 ± 755.45 | | 0.6698 |
|  |  |  | |  | |  |
| **IL7** |  |  | |  | |  |
| Baseline | 14 | 76.30 (43.02 – 121.38) | | 83.57 ± 55.35 | | N/A |
| 2 weeks | 14 | 95.60 (33.02 – 125.39) | | 99.71 ± 86.18 | | 0.9515 |
| 4 weeks | 14 | 106.79 (58.37 – 139.13) | | 101.53 ± 75.42 | | 0.6698 |
|  |  |  | |  | |  |
| **IL8** |  |  | |  | |  |
| Baseline | 14 | 9.45 (6.62 – 14.41) | | 13.37 ± 10.52 | | N/A |
| 2 weeks | 14 | 6.13 (5.19 – 9.17) | | 13.08 ± 21.82 | | 0.0785 |
| 4 weeks | 14 | 7.83 (5.62 – 15.99) | | 15.96 ± 22.51 | | 0.6698 |
|  |  |  | |  | |  |
| **MCP1** |  |  | |  | |  |
| Baseline | 14 | 250.15 (168.22 – 284.31) | | 241.19 ± 103.21 | | N/A |
| 2 weeks | 14 | 188.43 (138.39 – 279.00) | | 213.79 ± 86.51 | | 0.1937 |
| 4 weeks | 14 | 151.27 (121.15 – 246.85) | | 197.47 ± 102.73 | | 0.1531 |
|  |  |  | |  | |  |
| **MCSF** |  |  | |  | |  |
| Baseline | 14 | 0.90 (0.00 – 3.10) | | 2.36 ± 3.82 | | N/A |
| 2 weeks | 14 | 0.16 (0.00 – 1.88) | | 1.25 ± 1.81 | | 0.5566 |
| 4 weeks | 14 | 0.39 (0.00 – 3.18) | | 1.40 ± 1.75 | | 0.2402 |
|  |  |  | |  | |  |
| **MIG** |  |  | |  | |  |
| Baseline | 14 | 60.87 (29.90 – 108.78) | | 76.09 ± 58.59 | | N/A |
| 2 weeks | 14 | 67.24 (18.93 – 103.24) | | 83.64 ± 76.78 | | 0.7148 |
| 4 weeks | 14 | 52.65 (32.15 – 95.92) | | 61.72 ± 39.76 | | 0.2166 |
|  |  |  | |  | |  |
| **M1P1a** |  |  | |  | |  |
| Baseline | 14 | 66.92 (56.22 – 96.11) | | 83.46 ± 44.59 | | N/A |
| 2 weeks | 14 | 70.05 (48.97 – 82.32) | | 98.76 ± 102.15 | | 0.4631 |
| 4 weeks | 14 | 78.25 (57.59 – 158.11) | | 110.98 ± 87.37 | | 0.2412 |
|  |  |  | |  | |  |
| **M1P1b** |  |  | |  | |  |
| Baseline | 14 | 27.84 (20.39 – 44.22) | | 33.97 ± 19.07 | | N/A |
| 2 weeks | 14 | 24.38 (14.98 – 28.73) | | 25.58 ± 13.25 | | 0.0676 |
| 4 weeks | 14 | 21.51 (17.13 – 27.30) | | 31.24 ± 27.10 | | 0.3910 |
|  |  |  | |  | |  |
| **M1P1d** |  |  | |  | |  |
| Baseline | 14 | 311.86 (261.73 – 361.79) | | 317.07 ± 79.49 | | N/A |
| 2 weeks | 14 | 311.70 (264.04 – 378.86) | | 314.85 ± 74.16 | | 0.9515 |
| 4 weeks | 14 | 321.74 (282.98 – 378.96) | | 322.61 ± 78.14 | | 0.7609 |
|  |  |  | |  | |  |
| **PDGFBB** |  |  | |  | |  |
| Baseline | 14 | 17867.74 (13119.12 – 19311.70) | | 16070.93 ± 5844.38 | | N/A |
| 2 weeks | 14 | 18435.06 (14473.48 – 19634.08) | | 16200.95 ± 5700.90 | | 0.9032 |
| 4 weeks | 14 | 17843.52 (14275.41 – 19692.36) | | 16569.01 ± 4238.09 | | 0.5416 |
|  |  |  | |  | |  |
| **RANTES** |  |  | |  | |  |
| Baseline | 14 | 5990.88 (4491.89 – 6208.89) | | 5437.49 ± 958.48 | | N/A |
| 2 weeks | 14 | 5861.30 (4668.14 – 6396.10) | | 5532.78 ± 100.70 | | 0.4263 |
| 4 weeks | 14 | 6038.47 (4590.66 – 6251.90) | | 5612.62 ± 861.82 | | 0.2412 |
|  |  |  | |  | |  |
| **TIMP1** |  |  | |  | |  |
| Baseline | 14 | 4152.17 (3994.23 – 4452.94) | | 4260.69 ± 370.80 | | N/A |
| 2 weeks | 14 | 4227.79 (3989.47 – 4687.84) | | 4337.58 ± 524.55 | | 0.4631 |
| 4 weeks | 14 | 4372.97 (4048.86 – 4986.30) | | 4444.93 ± 605.98 | | 0.1189 |
|  |  |  | |  | |  |
| **TIMP2** |  |  | |  | |  |
| Baseline | 14 | 5670.77 (4321.09 – 6056.25) | | 5285.24 ± 953.04 | | N/A |
| 2 weeks | 14 | 5649.19 (4828.95– 6105.32) | | 5395.46 ± 954.42 | | 0.6257 |
| 4 weeks | 14 | 5462.87 (4880.55 – 5882.53) | | 5321.52 ± 816.96 | | 1.0000 |
|  |  |  | |  | |  |
| **TNFR1** |  |  | |  | |  |
| Baseline | 14 | 7673.58 (6659.62 – 8087.39) | | 7395.10 ± 1028.30 | | N/A |
| 2 weeks | 14 | 7595.06 (6297.02 – 7826.80) | | 7249.66 ± 952.91 | | 0.5380 |
| 4 weeks | 14 | 6582.81 (5360.01 – 7752.24) | | 6596.53 ± 1377.47 | | 0.0906 |
|  |  |  | |  | |  |
| **TNFα** |  |  | |  | |  |
| Baseline | 14 | 3.89 (2.71 – 4.83) | | 3.91 ± 2.02 | | N/A |
| 2 weeks | 14 | 3.77 (2.11 – 5.56) | | 5.22 ± 5.21 | | 0.6698 |
| 4 weeks | 14 | 5.75 (3.60 – 7.13) | | 7.80 ± 8.74 | | 0.2166 |
|  |  |  | |  | |  |
| **TNFβ** |  |  | |  | |  |
| Baseline | 14 | 530.05 (414.55 – 629.57) | | 552.21 ± 254.83 | | N/A |
| 2 weeks | 14 | 433.72 (252.57 – 638.11) | | 460.28 ± 326.69 | | 0.2676 |
| 4 weeks | 14 | 542.80 (286.38– 722.07) | | 528.63 ± 312.75 | | 0.7609 |
|  |  |  | |  | |  |
| **TNFR2** |  |  |  |  |  |  |
| Baseline | 14 | 7200.44 (6309.92 – 8558.88) | | 7342.40 ± 1234.32 | | N/A |
| 2 weeks | 14 | 7279.81 (7151.04 – 8056.21) | | 7440.55 ± 950.91 | | 0.8552 |
| 4 weeks | 14 | 6840.69 (6009.19 – 8186.78) | | 6830.27 ± 1403.87 | | 0.1937 |
|  |  |  | |  | |  |
| P value was evaluated by Wilcoxon signed-rank test (compared to baseline);  * Significance shown at p<0.05 | | | | | | |
