# Supplementary material for: Association of serum levels of inflammatory cytokines with retinopathy of prematurity in preterm infants
Source: Front Pediatr. 2024 Jan 8;11:1195904. doi: 10.3389/fped.2023.1195904 (PMC10800500; doi:10.3389/fped.2023.1195904)
Supplement: Supplementary file 4 [file Table4.docx]

| **Supplementary Table 4. Serum Inflammatory Cytokines Changes from Baseline up to 4 Weeks in No ROP (N = 14)** | | | | | | |
| --- | --- | --- | --- | --- | --- | --- |
| **Time** | **N** | **Median (Range), pg/mL** | | **Mean ± SD, pg/mL** | | **P value** |
| **BLC** |  |  |  |  |  |  |
| Baseline | 14 | 6.72 (4.68– 9.82) | | 7.26 ± 3.65 | | N/A |
| 2 weeks | 14 | 6.92 (5.67– 9.05) | | 8.84 ± 8.34 | | 0.5416 |
| 4 weeks | 14 | 8.56 (5.27– 15.13) | | 13.08 ± 13.71 | | 0.0580 |
|  |  |  | |  | |  |
| **Eotaxin** |  |  |  |  |  |  |
| Baseline | 14 | 338.80 (216.53 – 393.10) | | 313.61 ± 146.36 | | N/A |
| 2 weeks | 14 | 224.87 (145.44 – 355.83) | | 250.21 ± 128.78 | | 0.2676 |
| 4 weeks | 14 | 285.25 (186.30 – 359.74) | | 262.80 ± 109.10 | | 0.3258 |
|  |  |  | |  | |  |
| **Eotaxin2** |  |  |  |  |  |  |
| Baseline | 14 | 314.33 (123.83 – 379.45) | | 276.24 ± 161.90 | | N/A |
| 2 weeks | 14 | 184.06 (109.40 – 344.23) | | 229.09 ± 144.47 | | 0.5416 |
| 4 weeks | 14 | 249.74 (161.44 – 330.77) | | 255.78 ± 131.00 | | 0.5416 |
|  |  |  | |  | |  |
| **GCSF** |  |  |  |  | |  |
| Baseline | 14 | 17.73 (5.24– 26.97) | | 19.48 ± 16.89 | | N/A |
| 2 weeks | 14 | 10.91 (5.47 – 24.92) | | 24.57 ± 46.34 | | 0.6698 |
| 4 weeks | 14 | 11.60 (4.37 – 30.68) | | 25.93 ± 34.43 | | 0.8552 |
|  |  |  | |  | |  |
| **GMCSF** |  |  |  |  |  |  |
| Baseline | 14 | 46.43 (9.75 – 63.80) | | 51.11 ± 57.85 | | N/A |
| 2 weeks | 14 | 43.11 (31.10 – 59.27) | | 46.86 ± 30.59 | | 1.0000 |
| 4 weeks | 14 | 38.37 (14.42 – 60.27) | | 52.55 ± 55.28 | | 0.7148 |
|  |  |  | |  | |  |
| **I309** |  |  |  |  |  |  |
| Baseline | 14 | 0.90 (0.00 – 16.70) | | 14.70 ± 27.73 | | N/A |
| 2 weeks | 14 | 0.00 (0.00 – 8.41) | | 4.79 ± 8.39 | | 0.2754 |
| 4 weeks | 14 | 0.00 (0.00 –17.66) | | 16.72 ± 34.72 | | 0.5771 |
|  |  |  | |  | |  |
| **ICAM** |  |  |  |  |  |  |
| Baseline | 14 | 3115.91 (2609.25 – 3303.06) | | 2952.28 ± 615.10 | | N/A |
| 2 weeks | 14 | 2951.35 (2727.46 – 3530.23) | | 2908.98 ± 626.84 | | 0.7148 |
| 4 weeks | 14 | 3097.31 (2595.47 – 3463.40) | | 2938.29 ± 626.84 | | 0.9515 |
|  |  |  | |  | |  |
| **IF1a** |  |  |  |  |  |  |
| Baseline | 14 | 0.00 (0.00 – 0.24) | | 4.10 ± 12.94 | | N/A |
| 2 weeks | 14 | 0.00 (0.00 – 4.84) | | 3.98 ± 6.47 | | 0.3750 |
| 4 weeks | 14 | 0.00 (0.00 – 7.61) | | 14.16 ± 27.75 | | 0.2500 |
|  |  |  | |  | |  |
| **IF1b** |  |  |  |  |  |  |
| Baseline | 14 | 1.30 (0.02 – 2.14) | | 1.88 ± 2.21 | | N/A |
| 2 weeks | 14 | 0.66 (0.03 – 2.24) | | 1.85 ± 2.86 | | 0.7334 |
| 4 weeks | 14 | 2.18 (0.65 – 7.02) | | 4.21 ± 5.51 | | 0.3013 |
|  |  |  | |  | |  |
| **IFNg** |  |  |  |  |  |  |
| Baseline | 14 | 0.97 (0.42 – 2.16) | | 1.67 ± 1.79 | | N/A |
| 2 weeks | 14 | 1.36 (0.85 – 3.38) | | 9.14 ± 25.91 | | 0.1909 |
| 4 weeks | 14 | 1.44 (1.00 – 7.64) | | 15.60 ± 43.89 | | 0.1189 |
|  |  |  | |  | |  |
| **IL10** |  |  | |  | |  |
| Baseline | 14 | 13.12 (10.70 – 31.80) | | 24.41 ± 26.20 | | N/A |
| 2 weeks | 14 | 26.49 (10.29 – 31.46) | | 32.40 ± 38.31 | | 0.3910 |
| 4 weeks | 14 | 15.00 (10.90 – 59.05) | | 38.16 ± 42.17 | | 0.3258 |
|  |  |  | |  | |  |
| **IL11** |  |  | |  | |  |
| Baseline | 14 | 52.48 (23.14 – 75.01) | | 99.63 ± 168.47 | | N/A |
| 2 weeks | 14 | 65.97 (45.14 – 118.98) | | 78.46 ± 60.30 | | 1.0000 |
| 4 weeks | 14 | 42.80 (1.54 – 108.25) | | 73.52 ± 93.15 | | 0.6698 |
|  |  |  | |  | |  |
| **IL12p40** |  |  | |  | |  |
| Baseline | 14 | 11.48 (5.42 – 25.99) | | 14.79 ± 10.33 | | N/A |
| 2 weeks | 14 | 17.29 (9.02 – 25.63) | | 18.33 ± 10.99 | | 0.3910 |
| 4 weeks | 14 | 22.44 (13.63 – 27.73) | | 22.33 ± 12.42 | | **0.0419*** |
|  |  |  | |  | |  |
| **IL12p70** |  |  | |  | |  |
| Baseline | 14 | 0.20 (0.00 – 0.63) | | 0.41 ± 0.56 | | N/A |
| 2 weeks | 14 | 0.08 (0.00 – 0.46) | | 0.28 ± 0.40 | | 0.2783 |
| 4 weeks | 14 | 0.23 (0.00 – 0.86) | | 0.41 ± 0.48 | | 0.9697 |
|  |  |  | |  | |  |
| **IL13** |  |  | |  | |  |
| Baseline | 14 | 0.83 (0.57 - 1.43) | | 1.02 ± 0.78 | | N/A |
| 2 weeks | 14 | 1.00 (0.34 – 1.61) | | 1.48 ± 2.04 | | 0.8077 |
| 4 weeks | 14 | 0.69 (0.47 – 1.31) | | 2.07 ± 4.19 | | 0.5830 |
|  |  |  | |  | |  |
| **IL15** |  |  | |  | |  |
| Baseline | 14 | 0.99 (0.43 – 1.58) | | 1.21 ± 1.21 | | N/A |
| 2 weeks | 14 | 1.01 (0.38 – 2.27) | | 4.81 ± 13.40 | | 0.5016 |
| 4 weeks | 14 | 1.22 (0.62 – 3.17) | | 12.55 ± 36.50 | | **0.0295*** |
|  |  |  | |  | |  |
| **IL16** |  |  | |  | |  |
| Baseline | 14 | 168.13 (20.22 – 614.49) | | 313.00 ± 331.93 | | N/A |
| 2 weeks | 14 | 376.83 (218.62 – 539.19) | | 397.28 ± 291.04 | | 0.5016 |
| 4 weeks | 14 | 441.95 (202.47 – 773.60) | | 467.38 ± 340.58 | | 0.1726 |
|  |  |  | |  | |  |
| **IL17** |  |  | |  | |  |
| Baseline | 14 | 2.01 (0.68 – 6.18) | | 6.07 ± 12.86 | | N/A |
| 2 weeks | 14 | 2.75 (0.00 – 5.45) | | 5.09 ± 8.00 | | 0.5416 |
| 4 weeks | 14 | 3.64 (0.50 – 6.48) | | 6.84 ± 11.08 | | 0.3575 |
|  |  |  | |  | |  |
| **IL1ra** |  |  | |  | |  |
| Baseline | 14 | 0.40 (0.00 – 2.61) | | 1.17 ± 1.33 | | N/A |
| 2 weeks | 14 | 1.80 (0.00 – 2.29) | | 1.70 ± 2.00 | | 0.3594 |
| 4 weeks | 14 | 2.54 (0.00 -2.80) | | 3.39 ± 7.15 | | 0.2754 |
|  |  |  | |  | |  |
| **IL2** |  |  | |  | |  |
| Baseline | 14 | 13.63 (7.01 – 22.53) | | 19.77 ± 21.61 | | N/A |
| 2 weeks | 14 | 14.03 (8.28 – 24.68) | | 17.74 ± 12.31 | | 1.0000 |
| 4 weeks | 14 | 14.90 (11.01 – 22.43) | | 18.29 ± 13.43 | | 0.9515 |
|  |  |  | |  | |  |
| **IL4** |  |  | |  | |  |
| Baseline | 14 | 0.66 (0.00 – 3.03) | | 3.85 ± 8.17 | | N/A |
| 2 weeks | 14 | 2.29 (0.31 – 8.57) | | 6.36 ± 9.51 | | 0.2783 |
| 4 weeks | 14 | 4.52 (0.49 – 11.98) | | 10.24 ± 13.72 | | 0.0640 |
|  |  |  | |  | |  |
| **IL5** |  |  | |  | |  |
| Baseline | 14 | 7.28 (4.13 – 14.39) | | 10.92 ± 11.65 | | N/A |
| 2 weeks | 14 | 10.57 (5.76 – 15.47) | | 16.41 ± 22.65 | | **0.0353*** |
| 4 weeks | 14 | 9.84 (5.58 – 18.55) | | 24.07 ± 48.44 | | 0.2439 |
|  |  |  | |  | |  |
| **IL6** |  |  | |  | |  |
| Baseline | 14 | 5.32 (3.35 – 9.83) | | 7.38 ± 7.07 | | N/A |
| 2 weeks | 14 | 6.93 (3.83 – 11.60) | | 8.90 ± 7.25 | | 0.5016 |
| 4 weeks | 14 | 5.47 (3.44 – 8.72) | | 10.38 ± 16.55 | | 0.7609 |
|  |  |  | |  | |  |
| **IL6R** |  |  | |  | |  |
| Baseline | 14 | 4809.50 (4661.19 – 5280.28) | | 4918.09 ± 549.05 | | N/A |
| 2 weeks | 14 | 4902.28 (4325.17 – 5134.47) | | 4782.97 ± 537.01 | | 0.8552 |
| 4 weeks | 14 | 4951.64 (4637.82 – 5256.65) | | 4895.01 ± 577.46 | | 0.7609 |
|  |  |  | |  | |  |
| **IL7** |  |  | |  | |  |
| Baseline | 14 | 45.34 (20.08 – 62.76) | | 63.60 ± 69.86 | | N/A |
| 2 weeks | 14 | 50.22 (15.53 – 84.70) | | 57.72 ± 52.28 | | 0.8552 |
| 4 weeks | 14 | 46.94 (19.54 – 109.40) | | 76.04 ± 85.35 | | 0.6698 |
|  |  |  | |  | |  |
| **IL8** |  |  | |  | |  |
| Baseline | 14 | 6.10 (3.86 – 10.29) | | 8.51 ± 7.21 | | N/A |
| 2 weeks | 14 | 7.30 (4.02 – 14.01) | | 11.56 ± 11.11 | | 0.6257 |
| 4 weeks | 14 | 6.92 (3.49 – 15.35) | | 15.60 ± 27.35 | | 0.6257 |
|  |  |  | |  | |  |
| **MCP1** |  |  | |  | |  |
| Baseline | 14 | 278.10 (218.43 – 317.25) | | 265.64 ± 57.75 | | N/A |
| 2 weeks | 14 | 194.42 (134.27 – 322.87) | | 232.63 ± 100.42 | | 0.3575 |
| 4 weeks | 14 | 219.61 (150.04 – 252.01) | | 206.53 ± 62.21 | | 0.0785 |
|  |  |  | |  | |  |
| **MCSF** |  |  | |  | |  |
| Baseline | 14 | 0.12 (0.00 – 0.76) | | 1.49 ± 4.11 | | N/A |
| 2 weeks | 14 | 0.20 (0.00 – 1.88) | | 0.55 ± 0.98 | | 0.9219 |
| 4 weeks | 14 | 1.08 (0.00 – 3.27) | | 1.90 ± 2.49 | | 0.1475 |
|  |  |  | |  | |  |
| **MIG** |  |  | |  | |  |
| Baseline | 14 | 81.44 (12.79 – 127.56) | | 97.24 ± 104.09 | | N/A |
| 2 weeks | 14 | 65.36 (16.46 – 97.19) | | 100.62 ± 154.64 | | 0.9032 |
| 4 weeks | 14 | 91.01 (33.76 – 101.02) | | 98.25 ± 96.45 | | 0.8077 |
|  |  |  | |  | |  |
| **M1P1a** |  |  | |  | |  |
| Baseline | 14 | 65.39 (45.92 – 114.37) | | 81.99 ± 59.15 | | N/A |
| 2 weeks | 14 | 57.64 (29.70 – 81.72) | | 62.40 ± 35.33 | | 0.3575 |
| 4 weeks | 14 | 66.50 (47.66 – 83.94) | | 92.02 ± 89.94 | | 1.0000 |
|  |  |  | |  | |  |
| **M1P1b** |  |  | |  | |  |
| Baseline | 14 | 24.67 (13.75 – 56.04) | | 31.94 ± 25.27 | | N/A |
| 2 weeks | 14 | 18.48 (11.37 – 21.34) | | 19.78 ± 10.70 | | 0.1726 |
| 4 weeks | 14 | 21.99 (12.81 – 33.00) | | 24.10 ± 17.64 | | 0.6698 |
|  |  |  | |  | |  |
| **M1P1d** |  |  | |  | |  |
| Baseline | 14 | 291.24 (255.92 – 320.46) | | 283.26 ± 55.01 | | N/A |
| 2 weeks | 14 | 268.32 (231.21 – 325.79) | | 271.00 ± 76.65 | | 0.8552 |
| 4 weeks | 14 | 294.12 (239.07 – 345.39) | | 284.02 ± 76.64 | | 0.5016 |
|  |  |  | |  | |  |
| **PDGFBB** |  |  | |  | |  |
| Baseline | 14 | 13759.62 (5078.13 – 20406.75) | | 13439.77 ± 7672.71 | | N/A |
| 2 weeks | 14 | 14558.95 (10393.99 – 19256.36) | | 14526.25 ± 6063.04 | | 0.6698 |
| 4 weeks | 14 | 17593.47 (9922.92 – 22588.55) | | 16118.75 ± 6850.73 | | 0.1353 |
|  |  |  | |  | |  |
| **RANTES** |  |  | |  | |  |
| Baseline | 14 | 5440.21 (4763.22 – 6195.85) | | 5392.70 ± 1101.03 | | N/A |
| 2 weeks | 14 | 6056.54 (4393.0 – 6274.54) | | 5530.61 ± 1032.43 | | 0.9515 |
| 4 weeks | 14 | 5915.39 (4922.80 – 6784.01) | | 5726.25 ± 1075.89 | | 0.0906 |
|  |  |  | |  | |  |
| **TIMP1** |  |  | |  | |  |
| Baseline | 14 | 3991.75 (3841.30 – 4134.68) | | 4074.94 ± 373.90 | | N/A |
| 2 weeks | 14 | 3933.88 (3763.36 – 4169.84) | | 4007.61 ± 337.86 | | 0.6257 |
| 4 weeks | 14 | 4113.87 (3864.75 – 4265.37) | | 4095.89 ± 316.46 | | 0.5416 |
|  |  |  | |  | |  |
| **TIMP2** |  |  | |  | |  |
| Baseline | 14 | 4796.32 (4354.26 – 5776.50) | | 5003.85 ± 985.32 | | N/A |
| 2 weeks | 14 | 5074.92 (4037.01 – 5775.23) | | 5100.62 ± 988.43 | | 0.7148 |
| 4 weeks | 14 | 5473.35 (4685.56 – 5935.23) | | 5307.09 ± 841.03 | | 0.2166 |
|  |  |  | |  | |  |
| **TNFR1** |  |  | |  | |  |
| Baseline | 14 | 7546.71 (6202.51 – 7762.64) | | 7182.48 ± 1263.51 | | N/A |
| 2 weeks | 14 | 6935.91 (5770.69 – 7679.74) | | 6769.66 ± 1024.99 | | 0.2166 |
| 4 weeks | 14 | 6773.71 (6325.36 – 7421.68) | | 6828.12 ± 900.23 | | 0.3575 |
|  |  |  | |  | |  |
| **TNFα** |  |  | |  | |  |
| Baseline | 14 | 1.64 (0.85 – 3.65) | | 2.96 ± 3.59 | | N/A |
| 2 weeks | 14 | 1.87 (1.22 – 4.61) | | 3.50 ± 3.64 | | 0.3575 |
| 4 weeks | 14 | 2.40 (1.75 – 6.60) | | 6.09 ± 9.91 | | **0.0479*** |
|  |  |  | |  | |  |
| **TNFβ** |  |  | |  | |  |
| Baseline | 14 | 324.16 (215.19 – 634.85) | | 421.04 ± 383.94 | | N/A |
| 2 weeks | 14 | 265.29 (98.75 – 436.02) | | 335.07 ± 311.07 | | 0.5830 |
| 4 weeks | 14 | 315.92 (211.08 – 801.00) | | 455.27 ± 357.35 | | 0.6698 |
|  |  |  | |  | |  |
| **TNFR2** |  |  |  |  |  |  |
| Baseline | 14 | 7302.48 (6063.24 – 8191.01) | | 7102.67 ± 1416.08 | | N/A |
| 2 weeks | 14 | 7541.94 (5883.88 – 7908.38) | | 7144.44 ± 1368.90 | | 0.8552 |
| 4 weeks | 14 | 7042.34 (6512.13 – 7792.94) | | 6935.77 ± 1547.55 | | 0.8552 |
|  |  |  | |  | |  |
| P value was evaluated by Wilcoxon signed-rank test (compared to baseline);  * Significance shown at p<0.05 | | | | | | |
